# Mechanical Control of Whole Body Shape by a Single Cuticular Protein Obstructor-E in *Drosophila melanogaster*

**DOI:** 10.1371/journal.pgen.1006548

**Published:** 2017-01-11

**Authors:** Reiko Tajiri, Nobuhiro Ogawa, Haruhiko Fujiwara, Tetsuya Kojima

**Affiliations:** 1 Department of Integrated Biosciences, Graduate School of Frontier Sciences, The University of Tokyo, Kashiwa-shi, Chiba, Japan; 2 Laboratory Research Support Section, Center for Cooperative Research Promotion, Atmosphere and Ocean Research Institute, The University of Tokyo, Kashiwa-shi, Chiba, Japan; New York University, UNITED STATES

## Abstract

Body shapes are much more variable than body plans. One way to alter body shapes independently of body plans would be to mechanically deform bodies. To what extent body shapes are regulated physically, or molecules involved in physical control of morphogenesis, remain elusive. During fly metamorphosis, the cuticle (exoskeleton) covering the larval body contracts longitudinally and expands laterally to become the ellipsoidal pupal case (puparium). Here we show that *Drosophila melanogaster* Obstructor-E (Obst-E) is a protein constituent of the larval cuticle that confers the oriented contractility/expandability. In the absence of *obst-E* function, the larval cuticle fails to undergo metamorphic shape change and finally becomes a twiggy puparium. We present results indicating that Obst-E regulates the arrangement of chitin, a long-chain polysaccharide and a central component of the insect cuticle, and directs the formation of supracellular ridges on the larval cuticle. We further show that Obst-E is locally required for the oriented shape change of the cuticle during metamorphosis, which is associated with changes in the morphology of those ridges. Thus, Obst-E dramatically affects the body shape in a direct, physical manner by controlling the mechanical property of the exoskeleton.

## Author Summary

Shapes of objects, living or not, should depend on their material properties and forces acting on them. Mechanical processes that create whole body shapes of multicellular organisms, or genes that regulate such processes, are largely unknown. Insect bodies are coated by cuticle, a matrix composed of proteins and the polysaccharide chitin. We show that, during metamorphosis of the fruit fly *Drosophila melanogaster*, the cuticle covering the long and thin larva (maggot) undergoes longitudinal contraction and lateral expansion to become the short and stout puparium covering the pupa. Furthermore, we identify a single protein component of the larval cuticle that confers the oriented contractility/expandability, thereby determining the pupal body shape in a mechanical manner.

## Introduction

Development and evolution of biological forms have attracted people’s interest for centuries. Biologists have succeeded in elucidating how body plans—basic patterns of the body shared at the phylum level—are laid out by regulated expression of morphogens and transcription factors during development. Evidently, however, body shapes are significantly more variable than body plans: we note many examples of closely related species that differ dramatically in body shapes despite shared body plans, such as rhinoceroses and horses, or stick insects and leaf insects. These examples illustrate that there should be ways to change shapes without changing the basic patterns of the body.

Historically, there had been attempts at physical accounts of biological shapes, as in D’Arcy Thompson’s *On Growth and Form* [1], long before studies on the molecular mechanisms of body plan regulation. Modernized versions of such attempts probe into how constituent molecules regulate the physical properties, such as contractility or elasticity, of cells and extracellular matrices (ECM), and how those properties affect tissue shaping during development [2]. In considering whole body shapes, physical properties of ECM that constitute the animal skeleton, such as bones in vertebrates and exoskeleton (cuticles) in arthropods, should have a great impact, as they directly frame the bodies. Whether or how ECM molecules regulate body shapes through ECM mechanical properties remains elusive.

The insect cuticle is ECM that covers the entire body. It is generally composed of proteins and chitin (a polymer of N-acetyl-beta-D-glucosamine (GlcNAc)), both released from the underlying epidermis. On each sequenced insect genome, over 100 putative cuticular proteins are predicted, based on sequence similarity with known cuticular proteins [3]. A wide array of physical properties displayed by cuticles of different parts/stages/species is considered to be given by different combinations of cuticular proteins and their influences on the arrangement of chitin bundles (microfibrils) [4].

Here we show that loss-of-function mutations in *Drosophila melanogaster obstructor-E* (*obst-E*), a member of the conserved *obstructor* multigene family encoding putative cuticular proteins [5], causes body shape deformation at the level of the cuticle. During fly metamorphosis, the cuticle covering the final instar larval body (larval cuticle) directly converts into the puparium (pupal case) that covers the body through prepupal and pupal stages. In the normal metamorphosis of *D*. *melanogaster*, the body shape undergoes a dramatic change during puparium formation from the long and thin larval shape to the short and stout (rugby ball-like) pupal shape. By examining the changes in length and width of the cuticle, we show that the body shape change during puparium formation is mainly due to oriented shape change of the larval cuticle: contraction along the anteroposterior (longitudinal) axis and expansion in the lateral orientation. In *obst-E* mutants, the larval cuticle fails to undergo shape change and ends up as a twiggy, long and thin puparium. We further present evidence that Obst-E localizes to the larval cuticle, regulates the arrangement of chitin in the cuticle, and determines the pupal body shape by conferring oriented contractility/expandability on the cuticle. Thus, an ECM molecule greatly influences the body shape via direct regulation of ECM mechanical properties.

## Results

### “Twiggy” body shapes of the pupae, but not the larvae, of *obst-E* mutants

**Fig 1:**
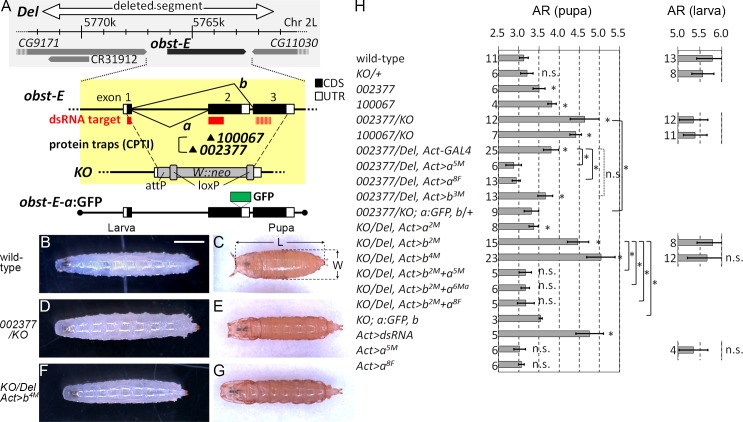
*obst-E* mutants show “twiggy” pupal shapes. (A) Schematic of the *obst-E* locus and mutations used in this study. Shaded in grey is the genomic structure around *obst-E*. The white arrow indicates the region deleted in *obst-E*^*Del*^. The gene structure and alternative splicing patterns of *obst-E* are magnified in the yellow shade. Black boxes are coding sequences and white boxes are untranslated regions. dsRNA is designed to target a portion of splice variant *a* mRNA that corresponds to the 35bp region at the 3’ end of exon 1 and the 468bp region at the 5’ end of exon 2 (red boxes). It is also expected to target exon 3 (red striped box), due to sequence similarity between exons 2 and 3. Arrowheads indicate locations of the CPTI insertions. In *obst-E*^*KO*^, the genomic region containing the entire coding sequences of both splice variants is replaced by an exogenous sequence. (B-G) Body shapes of larvae and pupae viewed from the dorsal sides. Anterior is to the left. Bar: 1mm. (H) Axial ratios (AR) of larvae and pupae of the indicated genotypes. The number of pupae/larvae measured for each genotype is shown in each graph. Error bars in this and all other figures are standard deviations. Asterisks with brackets indicate statistically significant difference (p<0.001). “n.s.” with a bracket, not significant difference. Asterisks/“n.s” without brackets, significant/not significant difference relative to wild-type.

During normal puparium formation of *D*. *melanogaster*, the long and thin larva turns into the rugby ball-shaped pupa (Fig 1B and 1C), as represented in the larval-to-pupal reduction in the body axial ratios (ARs, length/width) (Fig 1H). In the course of screening for morphological defects in mutants for genes encoding putative cuticular protein, we found that in homozygotes of protein trap insertions *obst-E*^*CPTI002377*^ and *obst-E*^*CPTI100067*^ (Fig 1A), the pupae appeared twiggier (longer and thinner) than those of the wild-type, as represented by larger ARs (Fig 1H). To analyze the function of *obst-E* in regulating the pupal body shape in more detail, we established two null alleles: the knockout allele (*obst-E*^*KO*^) and the deletion of four genes including *obst-E* (*obst-E*^*Del*^) (Fig 1A). The pupal shape phenotype was stronger in *obst-E*^*CPTI002377*^/*obst-E*^*KO*^ and *obst-E*^*CPTI100067*^/*obst-E*^*KO*^ heterozygotes than *obst-E*^*CPTI002377*^ and *obst-E*^*CPTI100067*^ homozygotes. The pupal body shapes were twiggy after metamorphosis, despite normal appearance in the larval stage (Fig 1D and 1E). The ARs of the final instar larvae of these *obst-E* mutants were comparable to those of the controls, but the pupal ARs of the mutants were significantly larger than those of the controls (Fig 1H). The twiggy pupal shape was phenocopied in flies in which RNAi-mediated knockdown of *obst-E* was induced throughout the body with the combination of *Actin*-GAL4 (*Act*-GAL4) and UAS-*obst-E* dsRNA (Fig 1H, see below). These observations indicate that the twiggy pupal shape is a loss-of-function phenotype of *obst-E*. It should be noted that the ARs are independent of variation in absolute body sizes (S1 Fig) [6]. Taken together, these observations show that *obst-E* function is required for efficient body shape change during metamorphosis.

Reduction of AR during normal metamorphosis could be due to decrease in body length (along the anteroposterior axis), increase in body width, or both. In wild-type and *obst-E*^*KO*^/+, the average body length decreased by 34% and 36%, while the average width increased by 22% and 10%, respectively. Thus, the AR reduction is associated with both longitudinal contraction and lateral expansion, with the former having a greater contribution. In *obst-E*^*CPTI002377*^/*obst-E*^*KO*^, the average body length decreased by only 12% and the average width increased by only 2% during metamorphosis, suggesting that changes in both orientations are impaired.

*obst-E* encodes two splice variants, denoted here as *a* and *b* (Fig 1A). Driving ubiquitous expression of variant *a* cDNA alone in the *obst-E*^*CPTI002377*^/*obst-E*^*Del*^ background using *Actin-*GAL4 fully rescued the pupal shape abnormality, while expression of variant *b* alone in the same background did not show significant rescue activity (Fig 1H). *obst-E* null mutants (*obst-E*^*KO*^ homozygotes and *obst-E*^*KO*^/ *obst-E*^*Del*^ heterozygotes) were first instar larval lethal. The larval lethality was efficiently rescued by driving ubiquitous expression of variant *b* using *Act*-GAL4 (hereafter referred to as *KO*/*Del*, *Act>b*), and the resultant pupae showed twiggy body shapes, despite normal larval body shapes (Fig 1F–1H). Expression of variant *a* in the null mutant background was much less efficient in rescuing the larval lethality (see below), but in occasional escapers, the pupal ARs were significantly smaller than those of the *KO*/*Del*, *Act>b* flies, although not as small as those of the controls (Fig 1H). Furthermore, variant *a*-specific knockdown by RNAi resulted in significantly larger pupal ARs (Fig 1H and S2 Fig). These results indicate that variant *a* is mainly responsible for the pupal shape regulation (see below). Because the pupal shape abnormality was most prominent in the *KO*/*Del*, *Act>b* flies, we mainly used them for further analysis of the function of *obst-E* in regulating metamorphic body shape change. The larval and pupal ARs of flies in which variant *a* was overexpressed in the wild-type background were comparable to those of the wild-type (Fig 1H), indicating that variant *a* is not capable of inducing excessive reduction of ARs beyond the wild-type level, either in the pupa or precociously in the larva.

It has been shown that, shortly before puparium formation, the anterior spiracles evert, followed by the retraction of the segments anterior to the spiracles (the pseudocephalon and the first thoracic segment), which brings the everted spiracles to the front of the body (Fig 1C, S3A and S3A’ Fig) [7][8]. In *obst-E* mutant prepupa, the anterior segments were normally internalized, but the anterior spiracles remained encapsulated within the puparium (Fig 1E and 1G, S3B and S3B’ Fig). These observations suggest that *obst-E* is dispensable for retraction of the larval head but is necessary for anterior spiracle eversion.

### Regulation of cuticle contraction by *obst-E*

**Fig 2:**
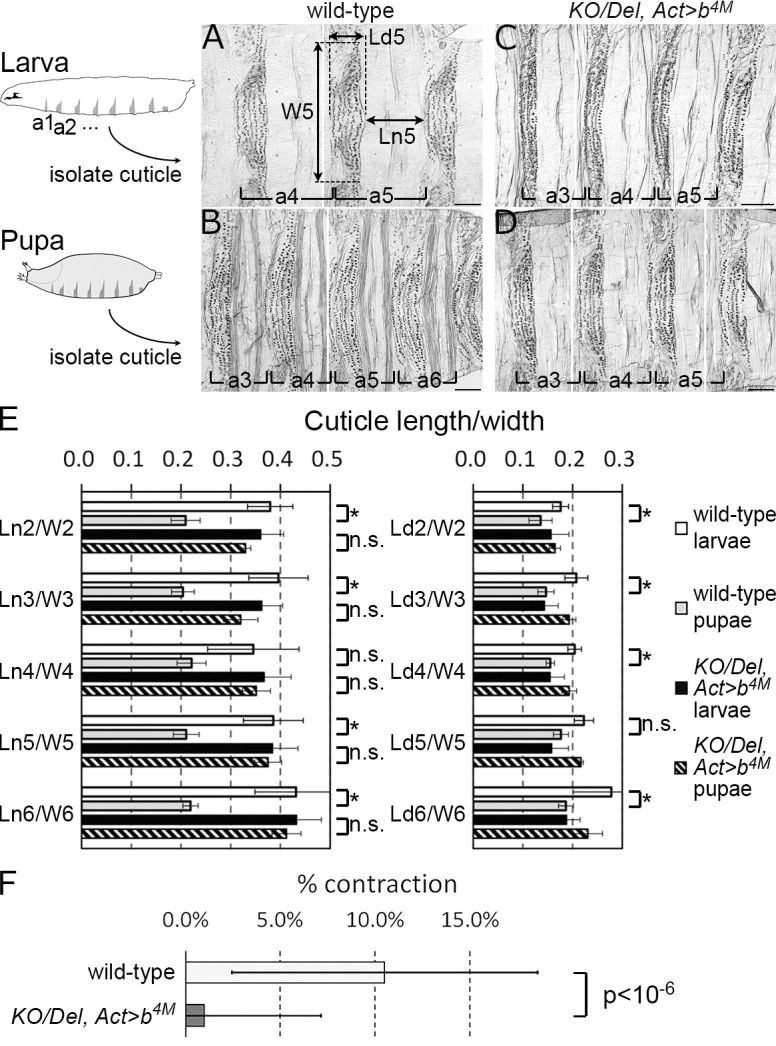
*obst-E* is required for shape change of the cuticle. (A-D) Ventral parts of isolated larval cuticles/puparia of wild-type (A, B) and *KO/Del*, *Act>b* (C, D). For each panel, 2–3 images are spliced (borders shown by white lines). Anterior is to the left. a, abdominal segment; W*k*, width of the denticle belt of *k*th abdominal segment; Ld*k*, length of the denticle belt; Ln*k*, length of the naked cuticle. Bars: 100 μm. (E) Averaged length/width ratios of cuticle in each denticle belt or naked region. The number of measured segments is shown in each graph. Asterisks, statistically significant difference (p<0.01, Student’s t-test); n.s., not significant. (F) Averaged contraction rates of cuticle in naked regions upon dehydration *ex vivo*. N = 33 naked regions for the wild-type and 40 naked regions for the mutant. p<10^−6^, Student’s t-test.

Generally in flies, the cuticle covering the final instar larval body becomes the puparium that covers the body through prepupal and pupal stages. To examine whether the *obst-E*-dependent mechanism of body shape change operates within the cuticle, isolated larval cuticles and puparia, from which all cellular tissues were removed, were observed. Using the segmentally repeated patterns of denticles (pigmented extensions on the larval cuticle that are preserved during metamorphosis) as landmarks, we analyzed how the length/width ratios of the cuticle of each segment changes during metamorphosis. In the wild-type, the length/width ratio of the cuticle dropped significantly during metamorphosis (Fig 2A, 2B and 2E), indicating that the cuticle *per se* underwent shape change. The cuticle shape change was especially prominent in regions devoid of denticles (naked regions). In contrast, in the *KO*/*Del*, *Act>b* larvae, which showed a severe defect in body shape change as described above, the cuticle did not show significant shape change during metamorphosis (Fig 2C–2E). These results indicate that the pupal shape abnormality of *obst-E* mutants results from reduced shape change of the larval cuticle itself during metamorphosis.

Fraenkel and Rudall showed that the blowfly larval cuticle lost water during metamorphosis, and that isolated larval cuticle exhibited significant contraction upon dehydration *ex vivo* [9]. We sought to address whether an equivalent dehydration-dependent mechanism in *D*. *melanogaster* can contribute to the difference between the wild-type and the *obst-E* mutant in degrees of cuticle contraction along the longitudinal axis during metamorphosis. Measurement using the wild-type *D*. *melanogaster* confirmed that the water content of the puparium was significantly lower than that of the final instar larval cuticle (S4 Fig). Isolated wild-type cuticle, from which cells are removed, contracted significantly along the longitudinal axis upon dehydration *ex vivo* (Fig 2F). In the *KO/Del*, *Act>b* mutant, the cuticle water content decreased significantly during puparium formation, as in the wild-type (S4 Fig). However, the isolated mutant larval cuticle did not show significant contraction along the longitudinal axis upon dehydration *ex vivo* (Fig 2F). Thus, *obst-E* regulates the physical property -contractility along the longitudinal axis in response to dehydration- of the larval cuticle.

### *obst-E*-directed formation of ridges in the third instar larval cuticle

**Fig 3:**
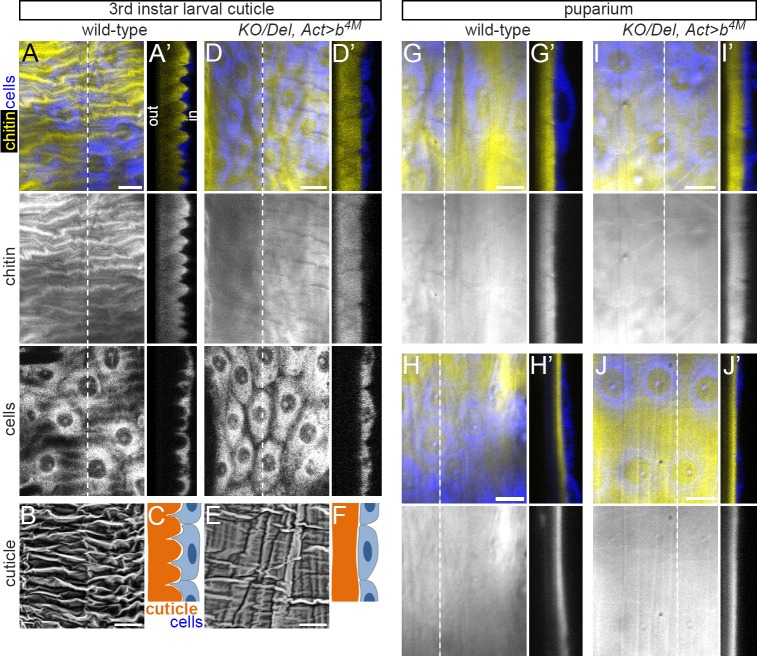
*obst-E* is required for the formation of ridge structure in the larval cuticle, which resolves during puparium formation. Three-dimensional structure of the late third instar larval cuticle of wild-type (A-C) and *KO/Del*, *Act>b* (D-F), and of the puparium of wild-type (G and H) and *KO/Del*, *Act>b* (I and J) at white prepupa (G and I) or 2 hours after white prepupa (H and J). A, D, G, H, I and J are projections of confocal images taken from the internal side. B and E are scanning electron micrographs of isolated larval cuticles taken from the internal side. A’, D’, G’, H’, I’ and J’ are optical cross-sections at the position of the dotted lines in A, D, G, H, I and J, respectively. C and F are schematic representations of A’ and D’, respectively. Anterior is to the left in A, B, D, E, G, H, I and J. External is to the left in A’, C, D’, F, G’, H’, I’ and J’. out, outer (external) surface of the cuticle; in, inner surface of the cuticle. Bars: 20 μm.

Close examination of the wild-type third (final) instar larval cuticle revealed that the inner surface of the cuticle was not smooth: it consisted of chitin-containing ridges that extended preferentially in the longitudinal orientation without respecting cell boundaries (Fig 3A–3C). Ridges were distributed throughout the inner surface of the wild-type third instar larval cuticle in a random manner without apparent association with subsegmental structures, except that the cuticle appeared flat in the pseudocephalon and at muscle attachment sites (S5 Fig). In contrast to the wild-type larval cuticle, the inner surface of the final instar larval cuticle of *KO*/*Del*, *Act>b* appeared essentially flat (Fig 3D–3F), except in the narrow lateral region where waving of the cuticle inner surface, albeit less prominent than the ridges in the equivalent regions of the wild-type cuticle, was observed (S5 Fig). In all subsequent presentation of cuticle cross-sections, dorsal or ventral regions (where ridges are formed on the third instar larval cuticle in a *obst-E*-dependent manner) are shown. The inner surface of the wild-type puparium observed just after puparium formation appeared flat (Fig 3G and 3G’), indicating that the ridges on the larval cuticle flattened during puparium formation. The thickness of the puparium subsequently decreased (Fig 3H and 3H’). In the *KO*/*Del*, *Act>b* mutant, the cuticle inner surface remained flat, and subsequent reduction in cuticle thickness occurred normally (Fig 3I, 3I’, 3J and 3J’). These observations suggest that the ridges on the wild-type third instar larval cuticle act as deformation units during puparium formation by generating contractile force in the longitudinal direction or expansive force in the orthogonal direction as they resolve.

**Fig 4:**
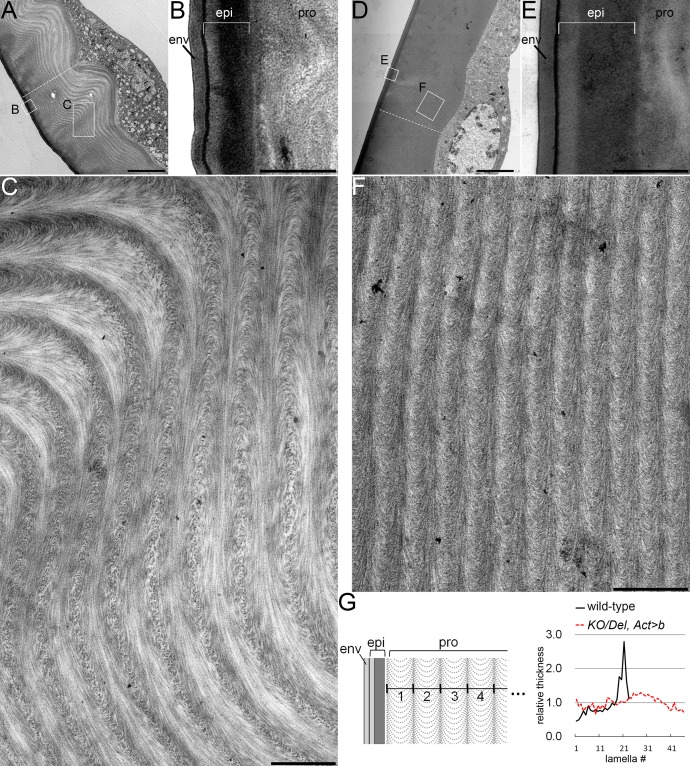
Electron micrographs of the wild-type and *KO/Del*, *act>b* third instar larval cuticle. Transmission electron micrographs of late third instar larval cuticle with the underlying epidermis of the wild-type (A-C) and of the *KO/Del*, *Act>b* mutant (D-F). Regions boxed in A and D are magnified in B, C, E and F. (G) Relative thickness of chitin lamellae. Lamella thickness was measured along the dashed white lines in A and D, and was normalized by the average lamella thickness. env, envelop; epi, epicuticle; pro, procuticle. Bars, 5 μm in A and D, 500nm in B, C, E and F.

The insect cuticle generally consists of three layers: the envelop (outer), the epicuticle (medial), and the procuticle (inner). The procuticle is a composite of chitin and proteins [10][11]. Chitin microfibrils in the procuticle are often arranged in the “twisted plywood” architecture, in which sheets of parallel microfibrils are piled up helicoidally along the apical-basal axis [12][13]. To investigate the structure of the *obst-E*-dependent ridges in more detail, we observed on a transmission electron microscope ultrathin sections of the final instar larval cuticle roughly perpendicular to the anterior-posterior axis. The general three-layer organization of the insect cuticle was observed in both the wild-type and the *KO*/*Del*, *Act>b* mutant. The outer two layers, the envelop and the epicuticle, appeared morphologically comparable between the wild-type and the mutant (Fig 4B and 4E). In the procuticle, although lamellae appeared as rows of stacked arcs, a characteristic appearance of the twisted plywood architecture of chitin microfibrils [12][13], both in the wild-type and in the mutant (Fig 4C and 4F), we found differences in their morphologies. In the wild-type, lamellae were thicker in the basal region (closer to the epidermal cells) than in the apical region (Fig 4A, 4C and 4G). Furthermore, lamellae were wavy, particularly in the basal region, and formed the overall ridge structure of the cuticle (Fig 4A and 4C). The convex and concave regions of the procuticle tended to be associated with alternating directions of chitin microfibril arcs within individual lamellae (Fig 4C and S6A Fig). In the procuticle of the *KO*/*Del*, *Act>b* mutant, lamellae were flat, and of relatively constant thickness, and tended to contain unidirectional arcs (Fig 4D, 4F and 4G and S6B Fig). These observations suggest that *obst-E* is involved in creating variations in the arrangement of chitin microfibrils that may cause the procuticle to buckle into the ridge structure, such as interchanging the direction of sheet rotation (manifested in alternating directions of arcs), and varying angles of sheet rotation or sheet thickness (manifested in varied lamella thickness) (see Discussion).

### Localization and chitin-binding activity of Obst-E-a protein

**Fig 5:**
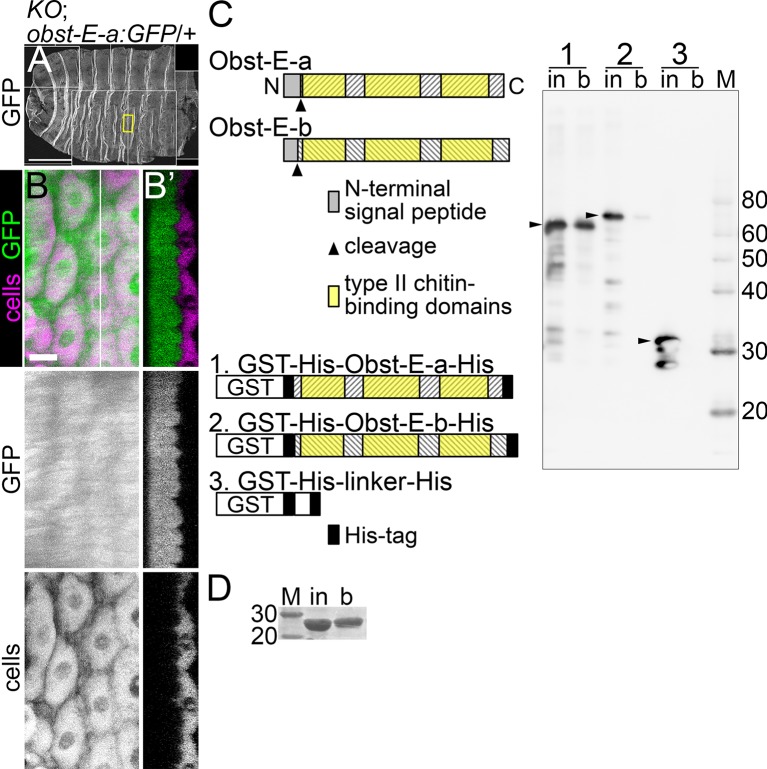
Obst-E localization and chitin-binding activity. (A,B) Localization of GFP signals in a late third instar larva which has a copy of *obst-E-a*:*GFP* in the *obst-E*^*KO*^ homozygous background. A is larval cuticle and epidermis of the whole body. The boxed region is magnified in B. B is a projection of confocal images taken from the internal side, and the optical cross-section at the position of the white line in B is shown in B’. Anterior is to the left in A and B, and external is to the left in B’. Bars, 500 μm in A, 20 μm in B. (C) Chitin-binding assay for the recombinant GST-His-Obst-E-a-His (1, 59kDa) and GST-His-Obst-E-b-His (2, 61kDa) proteins. GST-His-linker-His (3, 34kDa) was used as negative control. 1/400 of the input (1, 2) or 1/800 of the input (3) and 1/25 of the fraction bound to chitin beads (1, 2, 3) were loaded. in, input; b, bound. Arrowheads, bands corresponding to full-length proteins. (D) Chitin-binding assay for CBP. 1/4 of the input (in) and 3/4 of the bound fraction (b) were loaded. M, marker (kDa).

*obst-E* is a member of the *obstructor* multigene family encoding putative cuticular proteins with three type 2 chitin-binding domains (CBDs) [5]. Obstructor-A, encoded by another member of this family, was shown to bind chitin, and to co-localize with chitin on the epidermal surface [14]. To assess the distribution of Obst-E-a protein in vivo, we established an *obst-E-a*:*GFP* reporter, harboring a copy of the genomic sequence of *obst-E* into which the GFP sequence was inserted at the 3’ end of the coding sequence of variant *a* (Fig 1A). In the third instar larva having a copy of the transgene in either the wild-type or *obst-E*^*KO*^ homozygous background, GFP signals were detected uniformly throughout the cuticle including the ridges (Fig 5A, 5B and 5B’ and S7 Fig). The reporter significantly rescued the pupal shape abnormality of the *obst-E*^*CPTI002377*^/*obst-E*^*Del*^ mutant, and the presence of the reporter in the null mutant background resulted in significantly lower AR relative to the forced expression of variant *b* in the null background (Fig 1H). These results indicate that the GFP-tagged Obst-E-a protein expressed from the reporter was functional, at least to a certain extent.

To examine the chitin-binding activity of Obst-E proteins, we expressed and purified recombinant Obst-E-a and -b proteins, each tagged with GST and His tag, and tested whether they bound chitin beads. As a positive control, chitin-binding probe (CBP) bound to the beads (Fig 5D). Similarly, the tagged Obst-E-a protein was robustly detected in the fraction bound to the beads (Fig 5C), indicating that the protein had chitin-binding activity. Probing for tagged Obst-E-b in the bound fraction gave a faint signal (Fig 5C).

Taken together, these results suggest that Obst-E-a assembles with, and regulates the arrangement of, chitin to form oriented supracellular ridges in the extracellular space.

### Local requirement of *obst-E* for ridge formation

**Fig 6:**
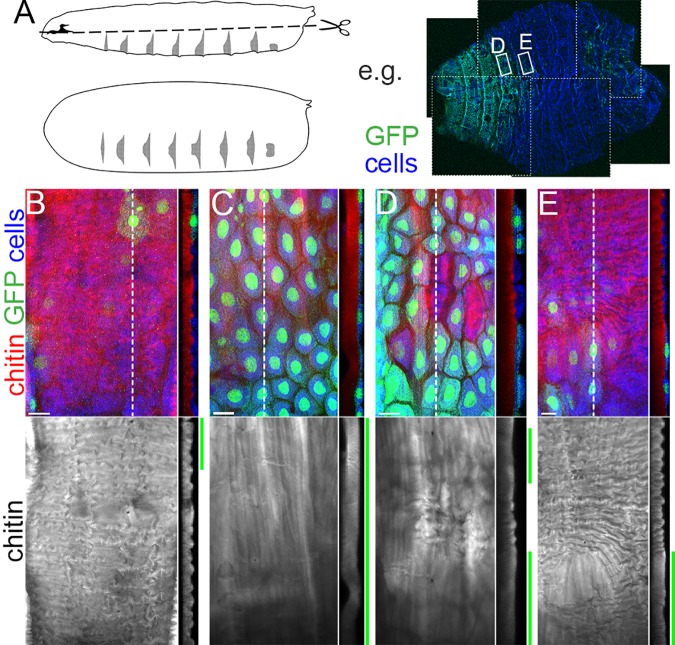
Obst-E locally directs ridge formation. (A) (Left) Preparation of larval cuticle and epidermis by cutting larvae open from the lateral sides. (Right) An example of cuticle and epidermis of late third instar larvae in which co-expression of *obst-E* dsRNA and GFP had been induced in mosaic manners, viewed from the internal side. Anterior is to the left. 6 images are spliced (borders shown by white dotted lines). (B-E) Structure of the larval cuticle relative to the distribution of GFP-positive cells in the underlying epidermis. The projection of confocal images taken from the internal side is shown on the left of each panel, and the optical cross-section at the position of the dotted line is shown on the right. Green bars indicate positions of GFP-positive cells in the cross-sections. Anterior is to the left in projections and external is to the left in cross-sections. Bars: 20 μm.

If Obst-E produced in the epidermis regulates formation of ridges in the overlying cuticle, knocking down *obst-E* in a subset of epidermal cells should result in local disruption of the ridge structure. To test this, we conducted mosaic RNAi analysis. In this experiment, the expression of double stranded RNA (dsRNA) against *obst-E* was induced in either the anterior or the posterior portion of larvae by locally heating larvae carrying a heat shock-inducible Flippase and an *Ay*GAL4 cassette (see Materials and Methods). dsRNA-expressing cells were labeled by co-expression of GFP. In regions containing no or sparse GFP-expressing epidermal cells, normal ridge structures were formed (Fig 6B). In regions consisting predominantly of GFP-expressing cells, the cuticle was essentially flat (Fig 6C). In mosaic regions containing both GFP-expressing and non-expressing cells, local disruption of the ridge structure, mainly over the GFP-expressing cells, was observed (Fig 6D and 6E). Thus, *obst-E* expression in the epidermis locally regulates ridge formation on the overlying cuticle. It should be noted that, although the disruption of ridge structure in the cuticle was strongly correlated to the distribution of GFP-expressing cells in the underlying epidermis, it was not strictly cell-autonomous: scattered GFP-positive cells tended to have little disruptive effect on the ridge structure (Fig 6B). Obst-E proteins secreted from the surrounding wild-type cells might have diffused into the cuticle over the GFP-positive cells and directed ridge formation there.

### Direct and local regulation of cuticle contractility and pupal shape by *obst-E*

**Fig 7:**
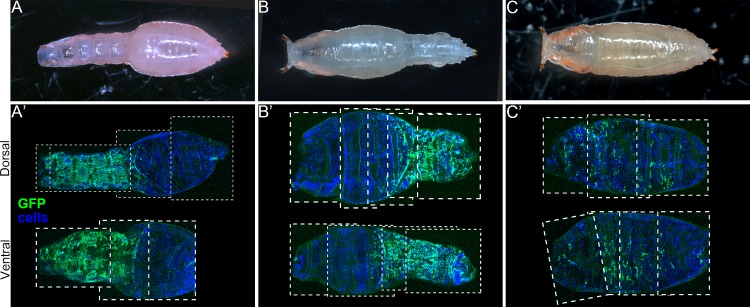
Local disruption of the ridge structure results in local deformation of the pupal shape. The pupal shapes (A-C) and the distribution of GFP-positive cells in the epidermis (A’-C’) are shown for seven individuals. All cells (GFP-positive and -negative) are stained by propidium iodide. Anterior is to the left. In A’, the epidermal cells in the anterior part of the body are mostly GFP-positive. In B’, the epidermal cells in the posterior part of the body are mostly GFP-positive. In C’, GFP-positive cells are sparse.

If the larval cuticular ridges are responsible for generating longitudinal contractile force and/or lateral expansive pressure that causes larval-to-pupal body shape change, local disruption of the ridge structure should result in local deformation of the pupal shape. To test this, larvae in which co-expression of *obst-E* dsRNA and GFP was induced in spatially restricted manners (as shown above) were reared until metamorphosis was complete. The resulting pupal shapes were recorded, and distribution of GFP-expressing cells in the epidermis was examined *a posteriori* by subsequently dissecting the pupae. As shown in Fig 7A and 7B, the pupae appeared twiggier specifically in regions where the epidermis consisted predominantly of GFP-expressing cells. Scattered GFP-positive cells, which would not affect the larval cuticular ridges (see above), did not affect pupal shapes (Fig 7C). These results support our hypothesis that *obst-E* expressed in the epidermis locally regulates formation of the oriented cuticular ridges, which in turn locally regulate oriented deformation of the cuticle. The local correlation between *obst-E* function and the pupal shape indicates direct causality from the function of Obst-E protein within the larval cuticle to the physical property of the cuticular ridges, and ultimately to the pupal body shape, rather than systemic effect of *obst-E* on metamorphosis or pupal morphogenesis.

### Temporal expression of *obst-E* and its function in early stages

**Fig 8:**
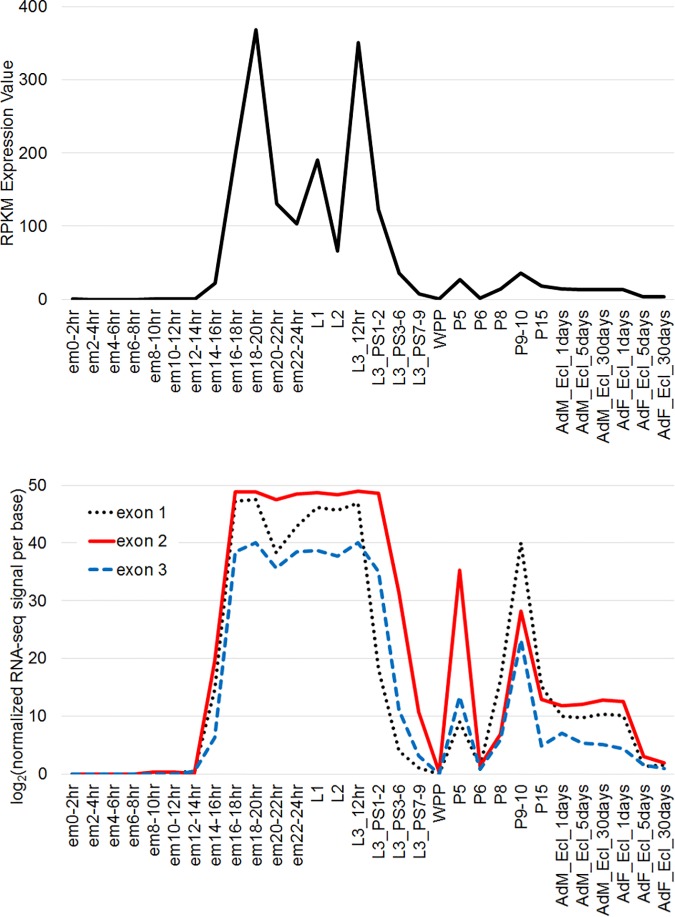
Temporal expression of *obst-E*. RNA expression profiles of the *obst-E* gene as a whole (upper) or of individual exons (lower) extracted from modENCODE developmental transcriptome data. em, embryo; L1, first instar larva; L2, second instar larva; L3, third instar larva; PS1-2, dark blue gut stage; PS3-6, light blue gut stage; PS7-9, clear gut stage; WPP, white prepupa; P, pupal stages; Ad, adult; M, male; F, female; Ecl, eclosion.

Temporal pattern of *obst-E* transcript level was extracted from modENCODE developmental transcriptome data (Fig 8). Expression of both variants rose sharply in the latter half of embryogenesis, persisted through first, second and early third instar larval stages, and decreased in the late third (wandering) larva. There appeared to be smaller peaks of expression during the prepupal and pupal periods.

**Fig 9:**
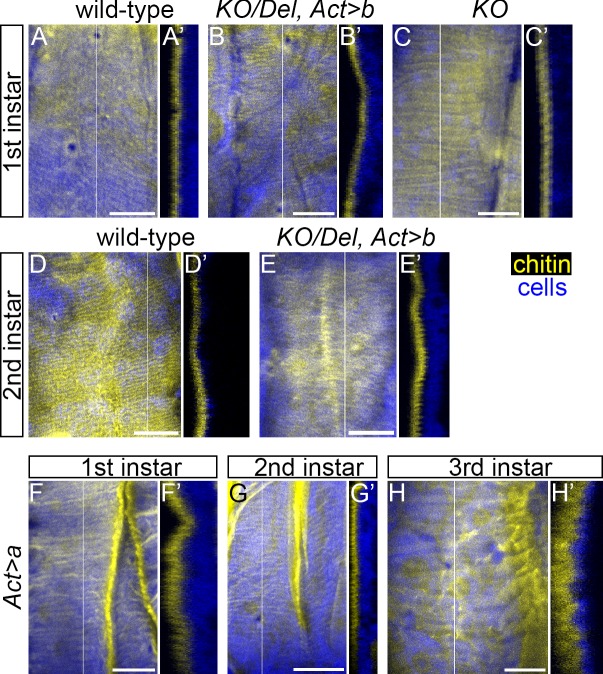
Loss of *obst-E* function does not affect early larval cuticle morphology, and overexpression of *obst-E-a* does not induce change in cuticle morphology. (A-C) First instar larval cuticle of wild-type (A), *KO/Del*, *Act>b* (B) and *obst-E*^*KO*^ homozygote (C). (D, E) Second instar larval cuticle of wild-type (D) and *KO/Del*, *Act>b* (E). (F-H) First instar (F), second instar (G) and third instar (H) larval cuticle of flies in which *obst-E-a* overexpression was induced with *Act*-GAL4. Chitin is in yellow, and cells are in blue. Optical cross-sections at the positions indicated by white lines in (A-H) are shown in (A’-H’). Anterior is to the left in projections and external is to the left in cross-sections. Bars, 20 μm.

Despite the expression of *obst-E-a* in stages earlier than the synthesis of the third instar larval cuticle, we did not find significant difference between the wild-type and the *KO*/*Del*, *Act>b* flies in the morphology of first and second instar larval cuticle. On the inner surface of the first instar larval cuticles of both the wild-type and the mutant, wavy patterns were barely observed (Fig 9A, 9A’, 9B and 9B’), while the inner surface of the second instar larval cuticles of both the wild-type and the mutant had fine wavy patterns (Fig 9D, 9D’, 9E and 9E’). In addition, overexpression of *obst-E-a* did not induce significant alteration in the morphology of the first, second or third instar larval cuticle (Fig 9F–9H). As mentioned above, *obst-E* null mutants were lethal during the first instar larval period. The cuticle of the first instar larvae of the null mutants appeared normal, both in integrity and in morphology (Fig 9C and 9C’ and S8A Fig). Meanwhile, in the first instar larvae of these null mutants, the hindguts protruded out of the anuses (S8A–S8D Fig), which probably caused the larval lethality. Mutant hindguts were morphologically normal during embryogenesis (S8E Fig). Thus, *obst-E* appears to be necessary for preventing hindgut protrusion after egg hatching.

As noted above, the pupal shape abnormality of *obst-E* mutants was rescued by the forced expression of variant *a*, but not by that of variant *b*. As to rescuing the larval lethality of *obst-E* null mutants, the forced expression of variant *b* was efficient, while that of variant *a* appeared less efficient (S1 Table). Simultaneous expression of the two variants rescued both the larval lethality and the pupal shape abnormality (Fig 1H). These results suggest that the two splice variants play distinct functions during development: variant *a* mainly regulates the pupal shape, and variant *b* mainly maintains the normal morphology of the larval hindgut.

### Evolution of the two variants of *obst-E*

**Fig 10:**
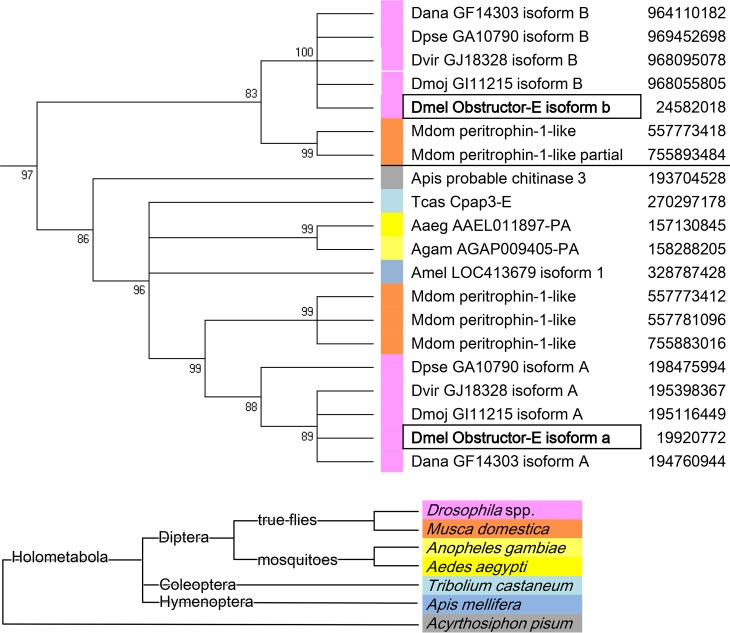
A phylogenetic tree of insect Obst-E proteins. The excerpt of the Obst-E group from the phylogenetic tree of Obst proteins shown in S9 Fig.

**Fig 11:**
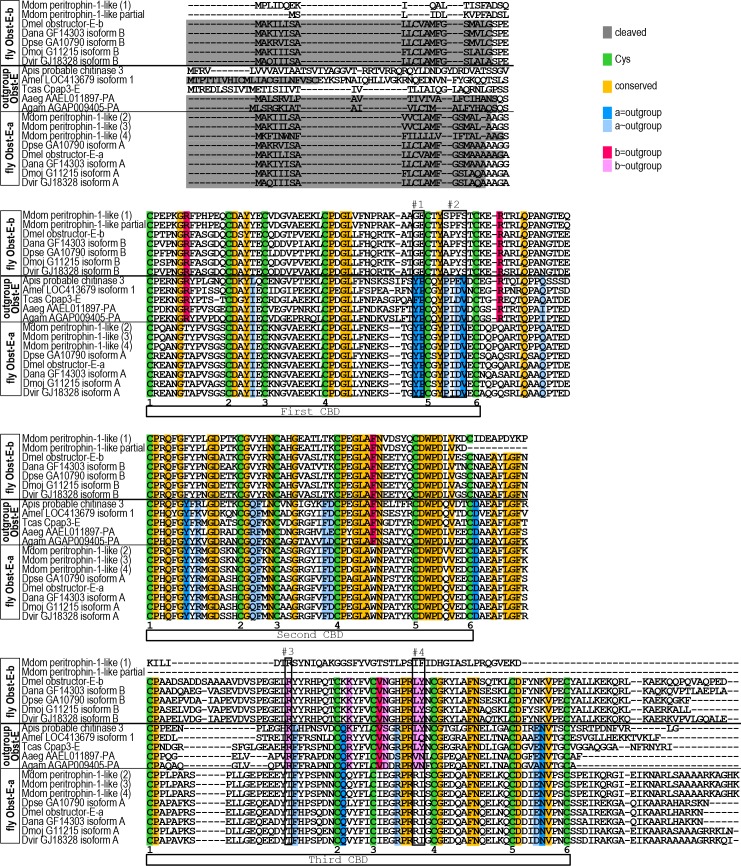
Alignment of Obst-E proteins. The three chitin-binding domains (CBDs) are indicated by white bars. Each contains 6 cysteines (green, numbered 1 to 6), typical of CBDs of Obst proteins, which probably form intradomain disulfide bonds [5]. Amino acids conserved across the Obst-E proteins of the outgroup species and the fly Obst-E variants a and b are shaded in orange. Amino acids strictly and moderately conserved in the fly Obst-E-a and the outgroup Obst-E proteins but not in the fly Obst-E-b proteins are shaded in blue and light blue, respectively. Amino acids strictly and moderately conserved in the fly Obst-E-b and the outgroup Obst-E proteins but not in the fly Obst-E-a proteins are shaded in magenta and light pink, respectively. Predicted signal peptides are in grey. #1–4, substitutions of amino acids with distinct properties (see text for detail). The NCBI GI numbers and RefSeq Accession numbers are: Mdom peritrophin-1-like (1) (557773418, XP_005186362.1), Mdom peritrophin-1-like partial (755893484, XP_005190169.2), Dmel obstructor-E-b (24582018, NP_723116.1), Dana GF14303 isoform B (964110182, XP_014761527.1), Dpse GA10790 isoform B (969452698, XP_015035293.1), Dmoj GI11215 isoform B (968055805, XP_015020857.1), Dvir GJ18328 isoform B (968095078, XP_015025340.1), Apis probable chitinase 3 (193704528, XP_001947458.1), Mdom peritrophin-1-like (2) (557773412, XP_005186359.1), Mdom peritrophin-1-like (3) (557781096, XP_005190168.1), Mdom peritrophin-1-like (4) (755883016, XP_011293693.1), Dpse GA10790 isoform A (198475994, XP_001357226.2), Dmel obstructor-E isoform A (19920772, NP_608957.1), Dana GF14303 isoform A (194760944, XP_001962692.1), Dmoj GI11215 isoform A (195116449, XP_002002767.1), Dvir GJ18328 isoform A (195398367, XP_002057793.1), Amel LOC413679 isoform 1 (328787428, XP_397120.4), Tcas Cpap3-E (270297178, NP_001161915.1), Aaeg AAEL011897-PA (157130845, XP_001662027.1)and Agam AGAP009405-PA (158288205, XP_310082.4). Full species names are listed in Fig 10 and in S9 Fig caption.

The functional divergence between the two splice variants of *D*. *melanogaster obst-E* prompted us to analyze how the gene evolved. Search for sequences homologous to *D*. *melanogaster obst-E* revealed that the existence of two variants was conserved within *Drosophila* and in the housefly *Musca domestica*. In contrast, mosquitoes (*Anopheles gambiae* and *Aedes aegypti*) and non-Dipteran insects seemed to possess a single form of *obst-E* (Fig 10 and S9 Fig, see Materials and Methods). In comparison of the full length proteins, the single Obst-E proteins of the mosquitoes and the non-Dipteran species showed higher homology to *D*. *melanogaster* variant a than to variant b (Fig 10 and S9 Fig). Upon closer inspection, we found some amino acid residues that were shared by the outgroup Obst-E and the fly Obst-E-a but not by the fly Obst-E-b, and conversely some that were shared by the outgroup Obst-E and the fly Obst-E-b but not by the fly Obst-E-a (Fig 11). Among them, substitutions of amino acids with distinct properties were found, such as (Y/F)P versus GE (Fig 11, #1), PX(D/E)V versus (S/A)X(F/Y)S (Fig 11, #2), R/K versus T (Fig 11, #3) and (L/V)(Y/Q/N) versus RI (Fig 11, #4). Those residues may differentiate the molecular functions of the splice variants and contribute to evolutionary divergence of their biological functions (see Discussion).

To further explore variation in the number of *obst-E* variants in Diptera, we made use of the recently published whole-genome shotgun sequences of Dipteran species [15] (see Materials and Methods). In four more ancestral species in the Brachycera, *Bactrocera oleae*, *Holcocephala fusca*, *Hermetia illucens*, and *Megaselis abdita*, two open reading frames, each showing homology to the three CBDs of *D*. *melanogaster* Obst-E-a and -b, were found. In *B*. *oleae*, *M*. *abdita* and *H*. *fusca*, the two were located tandemly on a single scaffold with a 0.1–1.1kb interval, while the two were found on separate scaffolds in *H*. *illucens* (S10 Fig). These results indicate that the presence of two *obst-E* variants is conserved in Brachycera. In contrast, only a single putative *obst-E* ortholog was found in Nematocera (non-Brachyceran Diptera) species, *Mayetiola destructor*, *Coboldia fuscipes* and *Tipula oleracea* (S10 Fig). The scaffolds on which the putative *obst-E* was located did not show any other homology to *D*. *melanogaster* Obst-E-a or -b. These results suggest that the Nematoceraspecies only possess a single form of *obst-E*, although we cannot rule out the possibility that the apparent lack of *obst-E* variants is simply due to the incompleteness of sequencing or sequence assembly.

## Discussion

We have shown that *Drosophila melanogaster* Obst-E is required for oriented deformation (longitudinal contraction and lateral expansion) of the larval cuticle into the rugby ball-shaped puparium. Obst-E is a structural constituent of oriented ridges within the larval cuticle. Mosaic analysis demonstrates that Obst-E expressed in the larval epidermis locally 1) directs the formation of the ridges in the overlying larval cuticle, and 2) confers oriented contractility/expandability on the cuticle so that it undergoes oriented deformation during puparium formation. Thus, Obst-E appears to determine the pupal body shape in a direct, mechanical manner by building supracellular “deformation units” that generate longitudinally contractile and laterally expansive forces during metamorphosis.

Fraenkel and Rudall suggested that body contraction along the longitudinal axis during metamorphosis in blowflies is aided by muscular processes [9][16]. Indeed, the *D*. *melanogaster tiggrin* mutants, in which structure and function of larval bodywall muscle are compromised, form uncontracted pupae [17]. In the present study, local correlation between epidermal *obst-E* expression and puparium deformation in the mosaic RNAi analysis, and the localization of Obst-E-a:GFP within the larval cuticle, indicate that Obst-E directly controls cuticle shape change within the cuticle itself. Buckling of the puparia in *obst-E* mutants (e.g. Figs 1G and 7) implies that bodywall muscles contract in these mutants. Formation of larva-like, uncontracted puparium has also been known in mutants in which hormonal induction of metamorphosis is compromised (e.g. [18]). The result of mosaic *obst-E* RNAi experiments rule out the possibility that *obst-E* regulates the shape of the puparium through systemic effect on metamorphosis. Although *obst-E* is dispensable for retraction of the anterior segments prior to puparium formation, it is necessary for eversion of the anterior spiracles. How Obst-E promotes the spiracle eversion is currently unclear.

How is the cuticle shape change driven at the molecular level? The larval cuticle also contains chitin, a water-insoluble GlcNAc polymer, together with a high content of water, which is reduced during metamorphosis. Although water-absorbing polymers generally swell and shrink upon hydration and dehydration, the *obst-E*-dependent cuticle contraction is unlikely to be the simple result of shrinkage upon dehydration, as metamorphic water reduction occurs even in the uncontracting *obst-E* mutant cuticle. Obst-E possesses three type 2 chitin-binding domains and shows chitin-binding activity (Fig 5C). Consistently, transmission electron micrographs of the third instar larval cuticle suggest roles of Obst-E in sculpting the chitin-containing layer, the procuticle, into the ridge structure, as well as in regulating how chitin microfibrils are arranged (Fig 4 and S6 Fig). Such Obst-E function may be required in the longitudinal contraction and lateral expansion of the cuticle during puparium formation. How the chitin microfibril arrangement and changes therein are related to the cuticle ridge structure, to the resolution of ridges during puparium formation, and ultimately to the whole body shape, is not yet known. In addition, the larval cuticle probably contains other cuticular proteins, which could also be involved in the cuticle remodeling. The exact mechanism by which the multiple cuticle components individually contribute to cuticle shape change needs to be addressed in the future.

As opposed to the existence of two Obst-E variants in the Brachyceran species tested, the existence of only a single form of Obst-E in non-Dipteran species, mosquitoes, and possibly in *Tipula*, *Coboldia* and *Mayetiola*, suggests that the two variants have arisen in an ancestor of the Brachycera. In Brachycera, puparium formation (direct conversion of the larval cuticle into the puparium) is a convergent feature of the Cyclorrhapha and the Stratiomyomorpha [19]. The existence of two Obst-E variants could have enabled, at some point in the evolution of Brachycera, employment of one of them for the regulation of pupal shape by controlling how the larval cuticle changes shape during puparium formation.

It is remarkable that a single constituent protein of the exoskeleton ECM can dramatically affect the shape of the whole body by regulating the physical property of the skeleton. Such “superficial” regulation of shape is in contrast to the widespread view associating body morphologies with expression patterns of morphogens and transcription factors. The physical aspects of morphogenesis have recently reemerged as a key to understanding biological shapes. For example, the formation of looping patterns in vertebrate gut tubes is explained physically by elasticity and relative growth of the gut tube and the anchoring tissue [20]. Identification of genes that control the physical aspects of morphogenesis will advance our understanding of how shapes are inherited and how they evolve. In this light, it is noteworthy that other exoskeleton components have recently been implicated in the regulation of tissue and body shapes. For example, some cuticular proteins are involved in the regulation of tracheal morphology and/or larval body shapes in *Drosophila* [6][21][22], and the control of the *Drosophila* adult wing shape requires the function of cuticle-related molecules, such as a transmembrane protein mediating the cell-cuticle attachment, an enzyme potentially involved in cross-linking of cuticle components, and genes specifically expressed during cuticle formation [23][24][25][26]. Changes in sequences or expression patterns of patterning genes (encoding morphogens and transcription factors) should often have pleiotropic effects that affect viability of the organism. Although changes in patterning genes could have driven significant morphological evolution in rare lucky cases that escaped fatal outcome, it may be difficult to account for frequent evolution of body shapes solely by changes in the patterning system. Evolution of ECM proteins may have been a key to driving frequent evolution of shapes by directly affecting the physical properties of ECM without affecting body patterning. In the case of *obst-E*, gene duplication appears to have been crucial for separating “essential” and “superficial” functions. Interestingly, insect genomes contain as many as two hundred genes encoding putative cuticular proteins, many of which have apparently arisen by gene duplication [6][27][28]. It would be interesting to see how functional divergence among the large number of cuticular proteins may have contributed to the diversity of shapes.

## Materials and Methods

### *Drosophila* genetics

Flies were reared on standard yeast-cornmeal food at 25°C. Canton-S and *w*^*1118*^ were used as wild-type. Cambridge Protein Trap Insertions (CPTI) 002377 and 100067 [29] were from the Kyoto Stock Center, UAS-*obst-E* dsRNA (11142R-1) was from the National Institute of Genetics, and *hsFLP*, UAS-*Dicer2* [30], *Actin5C*-GAL4 (*Act*-GAL4) and *Ay*GAL4, UAS-GFP.S65T [31] were from the Bloomington Stock Center. FRT-bearing PiggyBac insertions f04201 and f06711 from the Exelixis Collection (Harvard) were used to generate the deletion of four genes including *obst-E* (*obst-E*^*Del*^) by FLP-FRT-based method [32]. The deletion was verified by PCR using a primer pair flanking the deleted segment (Del Fw, 5’-TCGCAGTAGCTCTTCCTTAG-3’ and Del Rv, 5’-CTTAGTTCTTCCTGGCGTGA-3’) and a positive control pair located outside the deleted segment (Pc Fw, 5’-AACTGACCACCTTGGGTATG-3’and Pc Rv, 5’-GTTCTCGTTTCGCTCGTTTC-3’).

### Transgenic flies

For the UAS constructs, cDNAs for variants *a* and *b* of *obst-E* were subcloned from GH01453 and RE29976 of *Drosophila* Gene Collection [33] into pUAST using XhoI-SalI and EcoRI-KpnI, respectively. For the GFP-fusion construct, the entire *obst-E* gene region was first amplified by PCR using Canton-S genomic DNA as template and the following primers: *obst-E* Fw, 5’-AGGTCGACCTCGAGGCCACCACTCATAGGCAGTCA-3’ and *obst-E* Rv, 5’-AACGTTAACTCGAGGGTGGAAGTGGTTTGCCATCT-3’. The amplified fragment was cloned into StuI-digested pCaSpeR4 using In-Fusion HD Cloning Kit (Clontech) (pCaSpeR4-*obst-E*). The 3’ portion of variant *a* coding sequence (from the BglII site to the 3’ end) was amplified from pCaSpeR4-*obst-E*, and the EGFP sequence and the SV40 poly(A) signal was amplified from pPIG-A3GR [34], using the following primers: *a* Fw, 5’-GCCGGCACCCAGATCTGA-3’; *a* Rv, 5’-GCCCTTGCTCACCATGTTCTTCCTGGCGTGAAG-3’; GFP Fw, 5’-ATGGTGAGCAAGGGCGAGGA-3’; GFP Rv, 5’-CGAGCAGCTCAGATCCCAGGTTCTTCATTGGCTTC-3’. The two amplified fragments were cloned into BglII-digested pCaSpeR4-*obst-E* using In-Fusion HD Cloning Kit. Transgenesis was done by BestGene. For each UAS construct, several independent insertions (distinguished by superscript numericals) were used.

To assess how the forced expression of *obst-E* splice variants rescues larval lethality, crosses were set up between balanced *obst-E*^*Del*^, *Act*-GAL4 flies and balanced *obst-E*^*KO*^, UAS-*obst-E-a* or -*b* flies. Pupariated progenies were genotyped using the dominant markers on the balancers. The numbers of experimental progenies were normalized by the Mendelian ratio-adjusted numbers of sibling controls. For example, in a cross between *obst-E*^*KO*^*/GlaBc*; UAS-*obst-E-a*^*5M*^*/TM6b*. *Tb*^*1*^ and *obst-E*^*Del*^, *Act*-GAL4/*GlaBc*, the number of *obst-E*^*KO*^*/obst-E*^*Del*^, *Act*-GAL4; UAS-*obst-E-a*^*5M*^/+ (*Bc*^+^, *Tb*^+^) progenies was divided by [the number of *Bc*, *Tb*^+^ progenies]/2.

### Gene targeting

The *obst-E* knockout line was generated by homologous recombination-based gene targeting [35]. Briefly, 5’ and 3’ homology arms were amplified by PCR using the following primers: 5’ arm Fw, 5’-ACAGGGTAATGAATTCAGAGCATGGCTTACATAGAC-3’; 5’ arm Rv, 5’-TTGCATGCAAGAATTCCTAAGGAAGAGCTACTGCGA-3’; 3’ arm Fw, 5’-GAATCTGCAGCTCGAGTACCCGTACACTTCCTGTGC-3’; 3’ arm Rv, 5’-CCGACCTGCACTCGAGACTCATCTATCCGCAGGCAC-3’. The amplified 5’ and 3’ arms were cloned into the EcoRI site and the XhoI site, respectively, of pGX-attP-WN vector [35] using the In-Fusion HD Cloning Kit. Transgenesis was done by BestGene, and subsequent crosses were done as described in [35]. A single founder knockout line was established, and was subsequently verified genetically (heterozygotes with CPTI insertions exhibited twiggy pupal shapes) and by PCR using primers flanking the targeted region: KO Fw, 5’-CATTTTCGGCTGGCTGTTC-3’ and KO Rv, 5’-AAAGGAAACGGAAGCGATC-3’.

### Mosaic RNAi analysis

The local heat shock method from [36] was modified. Briefly, 5-10g of low melting point paraffin wax (Tm 46–48°C, Nacalai Tesque) was preheated until complete melting and was subsequently transferred to room temperature. When the wax surface began to solidify, anterior or posterior parts of anesthetized first instar larvae of the genotype *hsFLP*/Y or UAS-*Dicer2*; UAS-*obst-E* dsRNA / *obst-E*^*Del*^, *Ay*GAL4, UAS-GFP.S65T were pressed against the wax surface for 3–5 seconds. The larvae were immediately cooled in water, and were kept on standard food until the late third instar larval or the prepupal periods. Experiments with and without UAS-*Dicer2* gave comparable results.

### Measurement of body/cuticle length and width

Late third instar larvae were killed by placing them on a 65–70°C heat block for 10–30 seconds until movement ceased. Images of pupae and heat-killed larvae were taken from the dorsal sides with a Leica M165 FC stereo microscope, and were used to measure body lengths and widths on ImageJ (http://imagej.nih.gov/ij/). For cuticle isolation, the cuticles and the attached epidermis of pupae or heat-killed larvae were collected by manual dissection, and cellular tissues attached to cuticles were removed by incubation in 1% SDS for 1 hour. The isolated cuticles were washed, mounted in water, and imaged. The lengths and the widths of denticle belts and naked regions were measured on ImageJ. For cuticle dehydration *ex vivo*, cuticles were soaked in ethanol, air-dried, and imaged again. For each naked region, contraction rate was calculated as (Ln[wet]-Ln[dry])/Ln[wet].

### Cuticle weighing

Cuticles and puparia were isolated from late third instar larvae and white prepupae (up to ~1 hour after puparium formation), respectively, as described above. Cuticles/puparia of ~100 individuals were collected per test tube and weighed while wet on an electronic scale (Mettler Toledo AB104) three times each. The average of three weighings per tube was designated as wet weight. Cuticles/puparia were subsequently air-dried at 65–70°C for 1–2 hours and weighed three times. Water content for each tube was calculated as [wet weight]-[dry weight]/[wet weight].

### Staining and light microscopy

For histology of larval cuticle and epidermis, live late third instar larvae were dissected in PBS. The cuticle and epidermis was cut open and fixed in 4% formaldehyde/PBS for 10–30 minutes at room temperature. Cells were stained by propidium iodide (Thermo Fisher Scientific; 10 μg/ml), and chitin was stained by Fluostain (Calcofluor White Stain, Fluka; 2 μg/ml). Due to lack of RNase treatment, propidium iodide signals were found throughout the cytoplasm of larval epidermal cells. Muscles were manually removed before imaging by Olympus FV-1000.

For recording pupal shapes and GFP-positive cell distribution in mosaic RNAi flies, individuals were collected during white prepupal or early brown prepupal periods (up to ~3 hours after puparium formation). After body shapes were imaged as described above, prepupae were immediately dissected and fixed. Staining by propidium iodide and confocal imaging were done as described above.

For studying hindgut morphology, embryos were stained as previously described [37]. Anti-Fasciculin III antibody (7G10 [38], Developmental Studies Hybridoma Bank; 1:100) and Alexa Fluor 488-conjugated goat anti-mouse antibody (Thermo Fisher Scientific; 1:100) were used. First instar larvae were dissected in PBS, fixed in 4% formaldehyde/PBS for 10–30 minutes at room temperature, and stained with Alexa Fluor 647 Phalloidin (Thermo Fisher Scientific; 1:100) and Fluostain.

### Scanning electron microscopy

Cuticles were isolated from heat-killed larvae as described above, fixed in 2.5% glutaraldehyde/PBS for 1–2 hours at room temperature, dehydrated in ethanol and imaged by Hitachi Miniscope TM-1000. The average ridge width on the wild-type larval cuticle was 7.2±0.6 μm in the scanning electron micrographs and 10.7±1.2 μm in the confocal micrographs, suggesting that the dehydration procedure may have caused some shrinkage of the cuticle. However, the average cuticle width measured on the dorsal side of abdominal segment 5 in the scanning electron micrographs was 561±57 μm for wild-type and 582±47 μm for *KO/Del*, *Act>b*, suggesting that the dehydration procedure affected the wild-type and the mutant cuticle similarly.

### Sequence analysis

Similarity searches were done by tblastn (http://blast.ncbi.nlm.nih.gov/Blast.cgi) against RefSeq RNA sequences of the species listed in S9 Fig, using the full-length amino acid sequences of *Drosophila melanogaster* Obst-E-a and -b as queries. Full-length translations of all hits for variants a and b, together with *D*. *melanogaster* Obst-F~J, were subjected to multiple sequence alignments using Clustal Omega (http://www.ebi.ac.uk/Tools/msa/clustalo/). Based on this alignment, the phylogenetic tree in S9 Fig was generated. The Obst-E group is excerpted in Fig 10. The sequences of proteins of this group were aligned using Clustal Omega (Fig 11). Signal peptide cleavages were predicted by SignalP 4.0 [39].

In *Drosophila willistoni*, only the transcript corresponding to *Dmel obst-E-b* had been predicted for the *obst-E* ortholog GK15421 (FlyBase FB2016_01). In this case, tblastn search against the entire GK15421 gene region of the *D*. *willistoni* genome using *Dmel* Obst-E-a as the query revealed a high-scoring segment, probably corresponding to exon 2 of the *Dmel obst-E*, in the intron of the predicted gene. To eliminate the possibility that incomplete gene prediction or annotation of the *Anopheles* and *Aedes* genomes precluded identification of additional *obst-E* variants, the *obst-E* locus of these species were studied in more detail. For each species, the genome sequence of the *obst-E* locus flanked by neighboring genes was obtained from VectorBase (http://www.vectorbase.org, *Anopheles gambiae* PEST, AgamP3 and *Aedes aegypti* Liverpool, AaegL3), and was used as the subject for tblastn, with *Dmel* Obst-E-a and b as queries. For each species, high-scoring segments for both variants converged on the predicted CDS of the single *obst-E* ortholog.

To examine the number of *obst-E* variants in Dipteran species, tblastn search against the whole-genome shotgun contigs of the species listed in S10 Fig was conducted, using the full-length *D*. *melanogaster* Obst-E-a and -b as queries. The top three hits with the highest scores in each species were used as queries for reciprocal blastp search against *D*. *melanogaster* proteins, and those that gave either Obst-E-a or -b as the best match were considered as putative *obst-E* orthologs/variants. For each of *Bactrocera oleae*, *Megaselis abdita* and *Holcocephala fusca*, the top two hits met the criteria, and they were located on a single scaffold. In *Mayetiola destructor*, four hits, all on separate scaffolds, were found to meet the criteria. However, the four scaffolds extensively overlapped with high levels of nucleotide identity (96.1–99.9%) both within and outside of the hit regions, indicating that they form one continuous genomic region containing one putative *obst-E* ortholog, as shown in S10 Fig. In *Hermetia illucens*, the top two hits met the criteria for *obst-E* variants, but they were found on separate scaffolds. In *Coboldia fuscipes*, the top hit that met the criteria consisted of an ORF bearing homology to the first CBD of *D*. *melanogaster* Obst-E-a and -b, and an ORF with homology to the second and third CBDs of *D*. *melanogaster* Obst-E-a and -b, located tandemly on a single scaffold with a 67bp gap. In *Tipula oleracea*, the top hit that met the criteria for *obst-E* ortholog contained three segments: an ORF with homology to the first CBD, one with homology to the second CBD, and one with homology to the third CBD, of *D*. *melanogaster* Obst-E-a and -b. The three segments were separated by 85bp and 8bp gaps, respectively, and a stretch of unsequenced region was between the first and second segments. We consider the top hit in each of *C*. *fuscipes* and *T*. *oleracea* to represent the single *obst-E* ortholog. Frame inconsistencies between ORFs may be due to sequencing errors or insertion of introns.

### Chitin binding assay

Using full-length *obst-E-a* and *-b* cDNAs (see “Transgenic flies”) as templates, the coding sequences excluding the predicted N-terminal signal peptides were amplified using the following primers: a Fw, 5’-AGTCAGGAATTCTTTGGCTCAATGGCTGCC-3’; a Rv, 5’- AGTCGTCGACGTTCTTCCTGGCGTGAAG-3’; b Fw, 5’- TCAGTCAGTCGAATTCTTTGGCTCAATGGCTCTTGGC-3’ and b Rv, 5’- TCAGTCAGTCGTCGACATAGTCCTCCGGCTGA-3’. Each amplified fragment was digested with EcoRI and SalI and ligated into the pET41a(+) vector (Merck Millipore) digested with the same enzymes. For negative control, pET41a(+) was digested with BamHI and BglII and self-ligated. The resultant expression vectors, pET41a-GST-His-Obst-E-a-His, pET41a-GST-His-Obst-E-b-His and pET41a-GST-His-linker-His were transformed into BL21(DE3)pLysS strain (Merck Millipore). Protein expression was induced with IPTG according to the manufacturer’s instruction. Protein purification using glutathione beads was done using MagneGST Protein Purification System (Promega). Purified proteins were desalted using Microcon Centrifugal Filters (Merck Millipore), and were applied to Chitin Magnetic Beads (New England Biolabs) according to the manufacturer’s protocol. The fractions bound to the beads were collected by directly adding SDS-PAGE sample buffer to the beads and boiling. Samples were analyzed by SDS-PAGE, and western blotting was performed using Trans-Blot Turbo Transfer System and Trans-Blot Turbo Mini PVDF Transfer Packs (Bio-Rad). For immunodetection, a mouse anti-His antibody (GE Healthcare) was used at 1:3000 dilution, followed by reaction with an HRP-linked sheep anti-mouse IgG (GE Healthcare) at 1:10000 dilution. Chemiluminescence detection was done using ImmunoStar LD (Wako Pure Chemical Industries). As a positive control, chitin-binding probe (CBP) (New England Biolabs), a 26kDa protein containing the chitin-binding domain of Chitinase A1 from *Bacillus circulans* [40], was used. CBP was prepared in the laboratory of Shigeo Hayashi (RIKEN CDB) according to the protocol from Yinhua Zhang (New England Biolabs) [41]. CBP was detected by Coomassie Brilliant Blue staining of the SDS-PAGE gel.

### qPCR

Wild-type and *KO/Del*, *Act>b* mutant feeding larvae were collected at 91–94 hours after egg laying. Pools of larvae, each consisting of 8–12 larvae, were immediately homogenized in 500μl each of TRI reagent (Sigma-Aldrich), incubated for 5 minutes at room temperature, and stored at -70°C for 2–4 weeks. Total RNA was extracted according to the manufacture’s protocol. After DNaseI treatment (Takara) and phenol-chloroform extraction, reverse transcription was performed using Verso cDNA synthesis kit (Thermo Fisher Scientific) with random hexamer primers and 1μg of total RNA per pool as template. qPCR was conducted on StepOne (Thermo Fisher Scientific) using Power SYBR Green Master Mix (Thermo Fisher Scientific). The following primers were used. *obst-E* Fw (common), 5’-CGTTGCCATGTTTGGCTC-3’; *obst-E-a* Rv, 5’-AATGTAGGCATCGCAGGA-3’; *obst-E-b* Rv, 5’-GTGTAAGAGTCGCACTGG-3’; *RpL32* (*Ribosomal protein L32*) Fw, 5’-TACAGGCCCAAGATCGTGAAG-3’ and *RpL32* Rv, 5’-GACGCACTCTGTTGTCGATACC-3’. The specificity of all primers was verified by melt curve analysis. Standard curves were generated for all primer pairs, and relative expression levels of *obst-E* variants *a* and *b* were normalized using *RpL32* as an internal control.

### Transmission electron microscopy

Wild-type and *KO/Del*, *Act>b* mutant late third instar larvae were dissected in PBS, and the integuments (cuticle and epidermis) were fixed in 2.5% glutaraldehyde, 2% formaldehyde, PBS for 2 hours at room temperature. The fixed samples were embedded in 1% agarose in PBS and processed as described in [42], with small modifications. The embedded samples were postfixed with 1% OsO4 in 0.05M cacodylate buffer for 3 hours at room temperature, and then were dehydrated with a series of ethanol and propylene oxide. Subsequently, the samples were infiltrated and embedded in Epon812. Ultrathin sections approximately perpendicular to the longitudinal axis were stained with uranyl acetate and lead citrate, and were viewed on a JEOL JEM-1400 transmission electron microscope at 80kV.

### Temporal pattern of gene expression

The modENCODE developmental RNA-seq transcriptome profiles [43] for *obst-E* or for individual exons of *obst-E* were obtained via FlyBase (version FB2016_04).

## Supporting Information

S1 Fig Body axial ratios represent body shapes, regardless of body size variation. A scatter plot of body lengths versus widths of wild-type, *obst-E*^*KO*^/*+* and *obst-E*^*CPTI002377*^/*obst-E*^*KO*^ pupae. The dashed grey, blue and red lines represent L = W*(average axial ratio) for respective genotypes.
(TIF)

S2 Fig *obst-E-a* RNA is down-regulated by the expression of dsRNA. Relative expression levels of *obst-E-a* and *-b* in wild-type larvae and larvae in which the expression of dsRNA against *obst-E-a* is driven by *Act*-GAL4. p, Student’s t-test.
(TIF)

S3 Fig *obst-E-a* is involved in anterior spiracle eversion during puparium formation. The anterior region of wild-type (A, A’) and *KO/Del*, *Act>b* (B, B’) white prepupae. as, anterior spiracle; mh, mouth hook. Arrows indicate the retracted bodywall cuticle. Anterior is to the left. Bars, 50 μm.
(TIF)

S4 Fig Cuticle water content decreases during metamorphosis. Water contents of larval cuticles and puparia collected from late third instar larvae and white prepupae, respectively, of wild-type and *KO/Del*, *Act>b* flies are plotted. p, Student’s t-test.
(TIF)

S5 Fig Distribution of ridges on the inner surface of the third instar larval cuticle. (A-F) Scanning electron micrographs of the inner surface of wild-type (A-C) and *KO/Del*, *Act>b* (D-F) third instar larval cuticle. Arrows indicate muscle attachment sites. Anterior is to the left. (G-T) Confocal images of wild-type (G-P) and *KO/Del*, *Act>b* (Q-T) third instar larval cuticle. For G, H and O’, optical cross-sections at the white lines are shown on the right. Only optical cross-sections are shown in I, K-N and P-T. O is a Nomarski image of O’. Anterior is to the left in projections and external is to the left in cross-sections. t1-3, thoracic segments 1–3; a1-7, abdominal segments 1–7; mh, mouth hook; ci, cirri; asterisks, maxillary sense organs. Bars, 500 μm in A, D, J; 100 μm in B, C, E, F; 20 μm in G-I and K-T.
(JPG)

S6 Fig Arc directions in the third instar larval cuticle. Transmission electron micrographs of Wild-type (A) and *KO/Del*, *Act>b* (B) third instar larval cuticle. Arcs visible in the micrographs are represented as colored “C” shapes in the lower panels. Red and green indicate alternating directions of arcs. Bars, 2 μm.
(JPG)

S7 Fig Obst-E-a:GFP localization in the wild-type background. Cuticle of a third instar larva having a copy of the *obst-E-a*:GFP reporter in the wild-type background. Panels on the left show a projection of confocal images taken from the internal side, and the optical cross-section at the dashed white line is shown on the right. Bar, 20μm.
(TIF)

S8 Fig Hindgut protrusion in *obst-E* null mutant larvae. (A) A first instar *obst-E*^*KO*^/ *obst-E*^*Del*^ larva. (B, C) The posterior parts of first instar *obst-E*^*KO*^ homozygous larvae. Boxed regions are magnified in B’, C’ and C”. The gut musculature is visualized by phalloidin (green), and cuticle is stained by Fluostain (magenta). Arrowheads, anuses; arrows, doubled gut walls. (D) A model of how the hindgut protrudes to form the “doubled wall” morphology. Green, hindgut walls; magenta, cuticle. (E) Wild-type and *obst-E*^*KO*^ homozygous embryos stained by anti-Fasciculin III antibody. Hindguts are pseudocolored in cyan.
(TIF)

S9 Fig A phylogenetic tree of insect proteins homologous to *D*. *melanogaster* Obst-E proteins. Phylogenetic analysis was done by MEGA5 [44] using the JTT cost matrix and complete deletion of indels. Bootstrap support is based on 1000 resampled data sets. Branches corresponding to partitions reproduced in less than 80% bootstrap replicates are collapsed. Dmel, *Drosophila melanogaster*; Dana, *Drosophila ananassae*; Dpse, *Drosophila pseudoobscura*; Dmoj, *Drosophila mojavensis*; Dvir, *Drosophila virilis*; Mdom, *Musca domestica* (housefly); Agam, *Anopheles gambiae* (malaria mosquito); Aaeg, *Aedes aegypti* (yellow fever mosquito); Tcas, *Tribolium castaneum* (red flour beetle); Amel, *Apis mellifera* (European honey bee); Apis, *Acyrthosiphon pisum* (pea aphid). 8–9 digit numbers on the right are NCBI GI numbers.
(TIF)

S10 Fig Putative *obst-E* orthologs/variants in Dipteran species. (Left) The phylogenic tree of the species examined, according to [15] and [19]. (Middle) The scaffold numbers and GenBank IDs of genomic sequences on which putative *obst-E* orthologs/variants were found by reciprocal blast searches with *D*. *melanogaster*. (Right) The distribution of the blast hit(s) on each genomic scaffold.
(TIF)

S1 Table Efficient rescue of larval lethality by the expression of variant *b*, but not by that of variant *a*. Normalized survival rates to puparium formation of flies in which expression of either variant was induced by different UAS lines in the *obst-E*^*KO*^/ *obst-E*^*Del*^ background. The numbers in each parenthesis are (the number of experimental progenies / the Mendelian ratio-adjusted number of sibling controls).
(TIF)
