# Supplementary figures and images for: Mechanical Control of Whole Body Shape by a Single Cuticular Protein Obstructor-E in Drosophila melanogaster
Source: PLoS Genet. 2017 Jan 11;13(1):e1006548. doi: 10.1371/journal.pgen.1006548 (PMC5226733; doi:10.1371/journal.pgen.1006548)

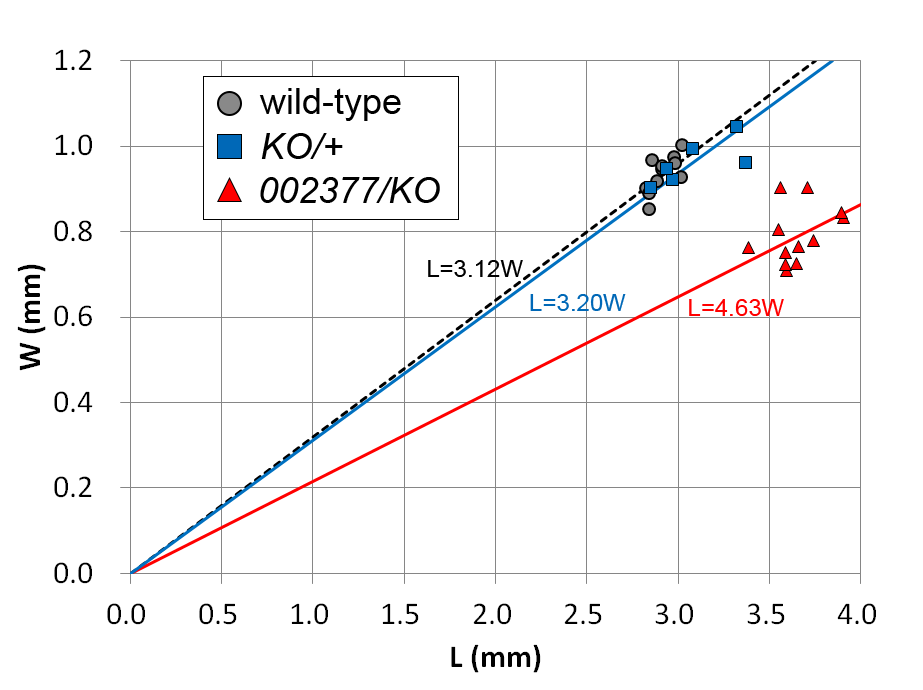

Supplement: S1 Fig — A scatter plot of body lengths versus widths of wild-type, obst-EKO/+ and obst-ECPTI002377/obst-EKO pupae. The dashed grey, blue and red lines represent L = W*(average axial ratio) for respective genotypes. (TIF) [file pgen.1006548.s001.tif]

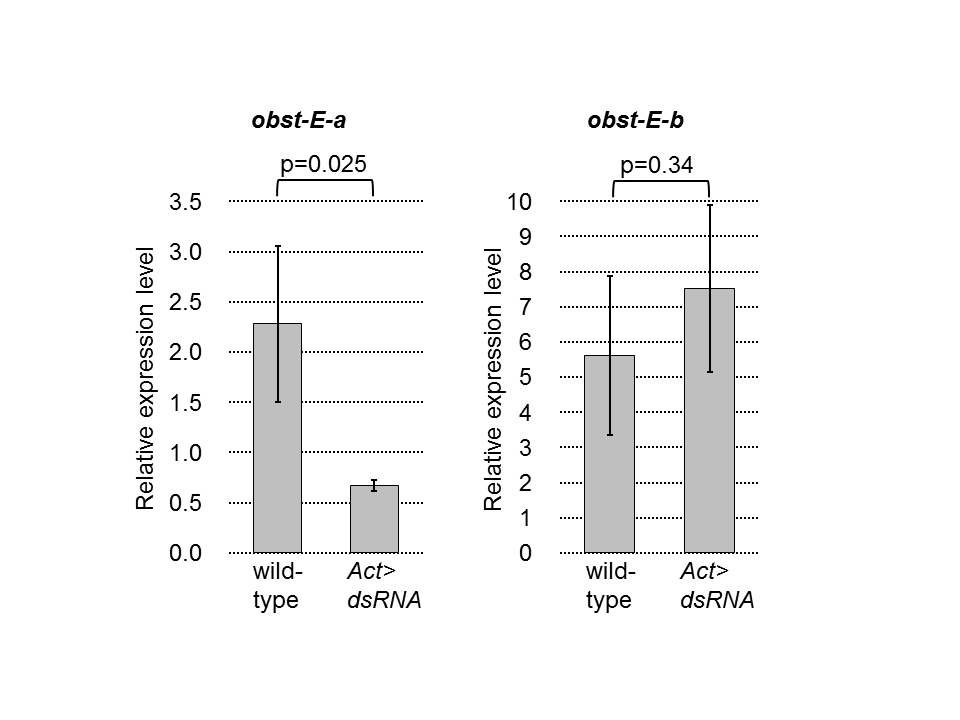

Supplement: S2 Fig — Relative expression levels of obst-E-a and -b in wild-type larvae and larvae in which the expression of dsRNA against obst-E-a is driven by Act-GAL4. p, Student’s t-test. (TIF) [file pgen.1006548.s002.tif]

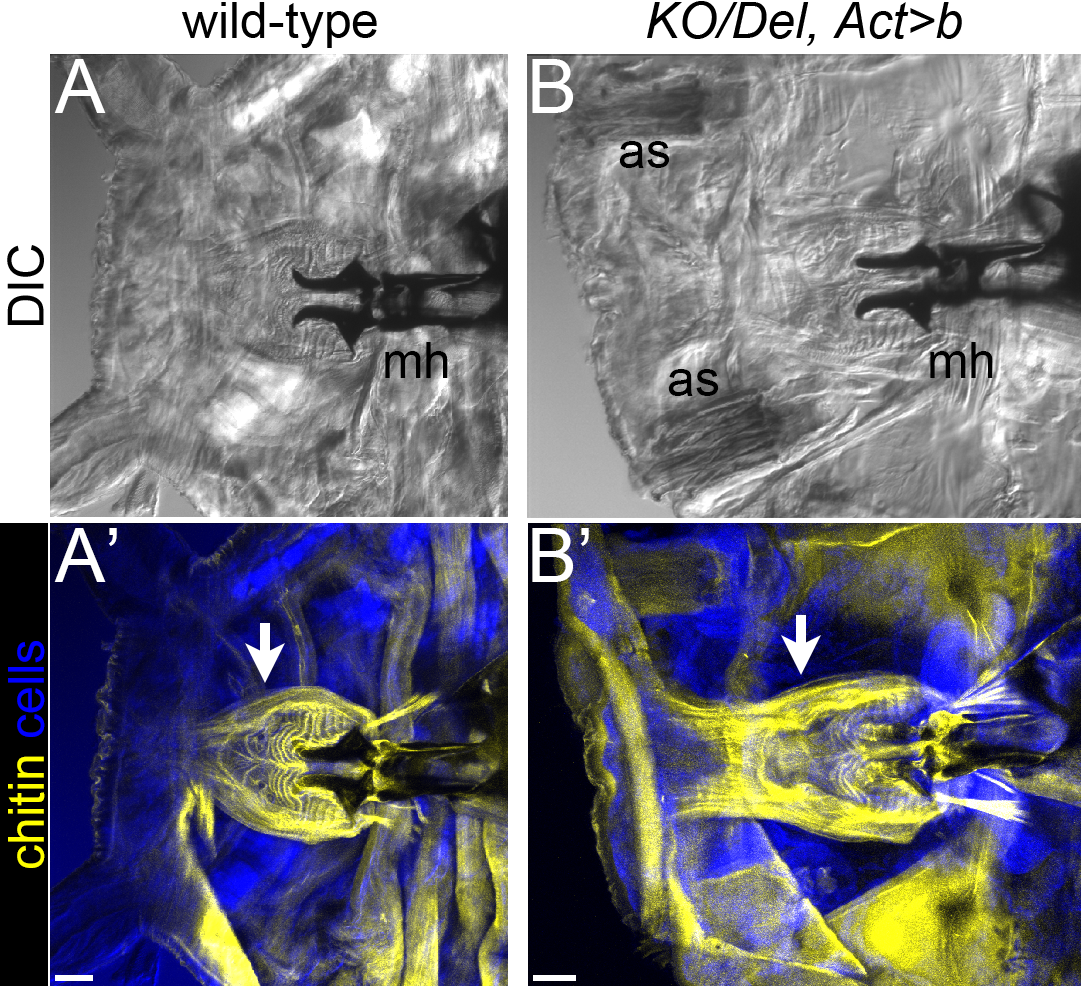

Supplement: S3 Fig — The anterior region of wild-type (A, A’) and KO/Del, Act>b (B, B’) white prepupae. as, anterior spiracle; mh, mouth hook. Arrows indicate the retracted bodywall cuticle. Anterior is to the left. Bars, 50 μm. (TIF) [file pgen.1006548.s003.tif]

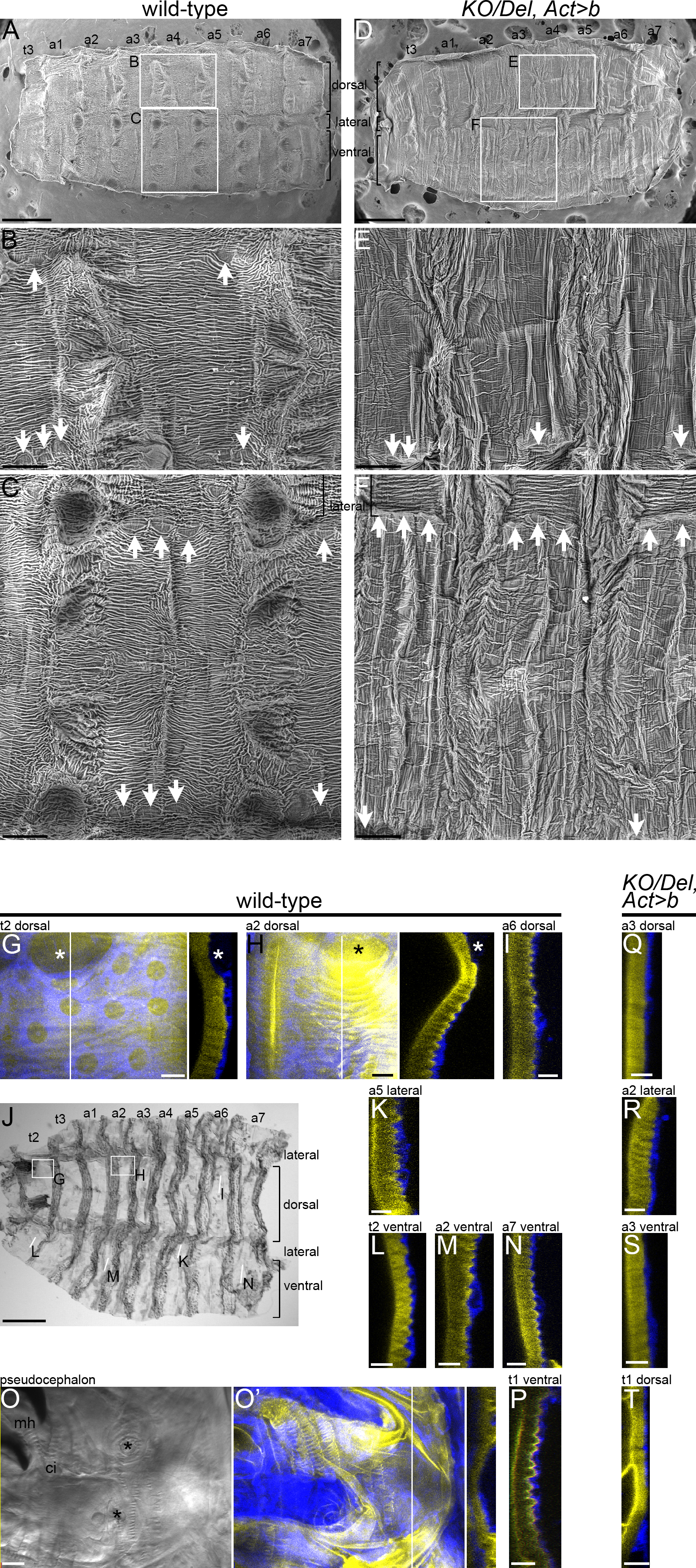

Supplement: S5 Fig — (A-F) Scanning electron micrographs of the inner surface of wild-type (A-C) and KO/Del, Act>b (D-F) third instar larval cuticle. Arrows indicate muscle attachment sites. Anterior is to the left. (G-T) Confocal images of wild-type (G-P) and KO/Del, Act>b (Q-T) third instar larval cuticle. For G, H and O’, optical cross-sections at the white lines are shown on the right. Only optical cross-sections are shown in I, K-N and P-T. O is a Nomarski image of O’. Anterior is to the left in projections and external is to the left in cross-sections. t1-3, thoracic segments 1–3; a1-7, abdominal segments 1–7; mh, mouth hook; ci, cirri; asterisks, maxillary sense organs. Bars, 500 μm in A, D, J; 100 μm in B, C, E, F; 20 μm in G-I and K-T. (JPG) [file pgen.1006548.s005.jpg]

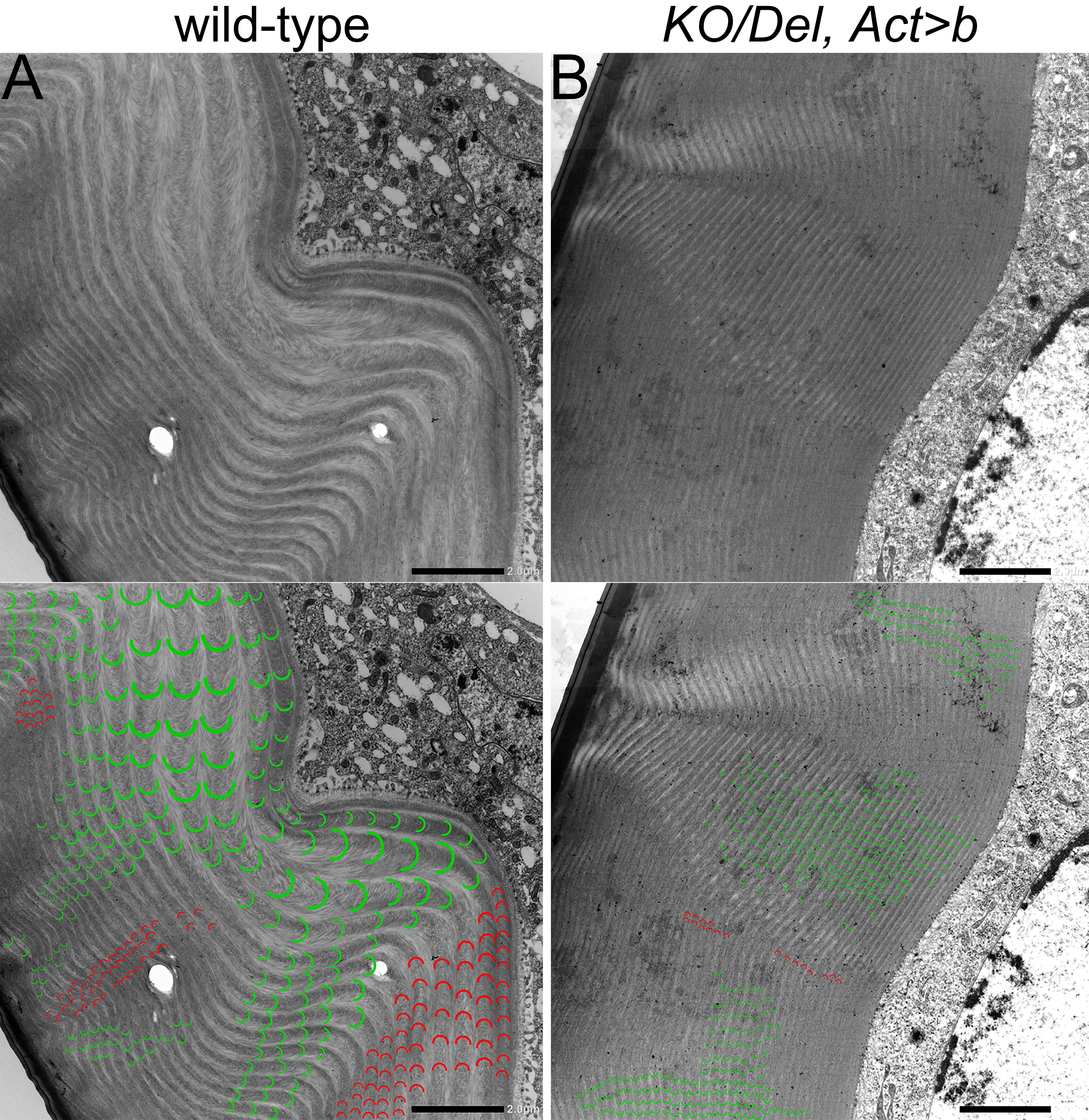

Supplement: S6 Fig — Transmission electron micrographs of Wild-type (A) and KO/Del, Act>b (B) third instar larval cuticle. Arcs visible in the micrographs are represented as colored “C” shapes in the lower panels. Red and green indicate alternating directions of arcs. Bars, 2 μm. (JPG) [file pgen.1006548.s006.jpg]

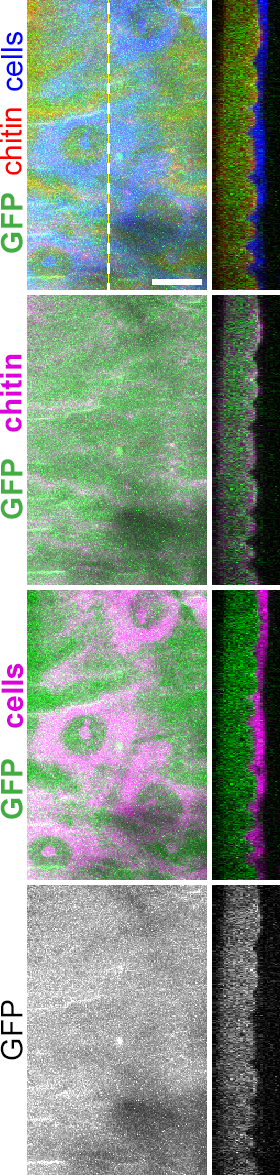

Supplement: S7 Fig — Cuticle of a third instar larva having a copy of the obst-E-a:GFP reporter in the wild-type background. Panels on the left show a projection of confocal images taken from the internal side, and the optical cross-section at the dashed white line is shown on the right. Bar, 20μm. (TIF) [file pgen.1006548.s007.tif]

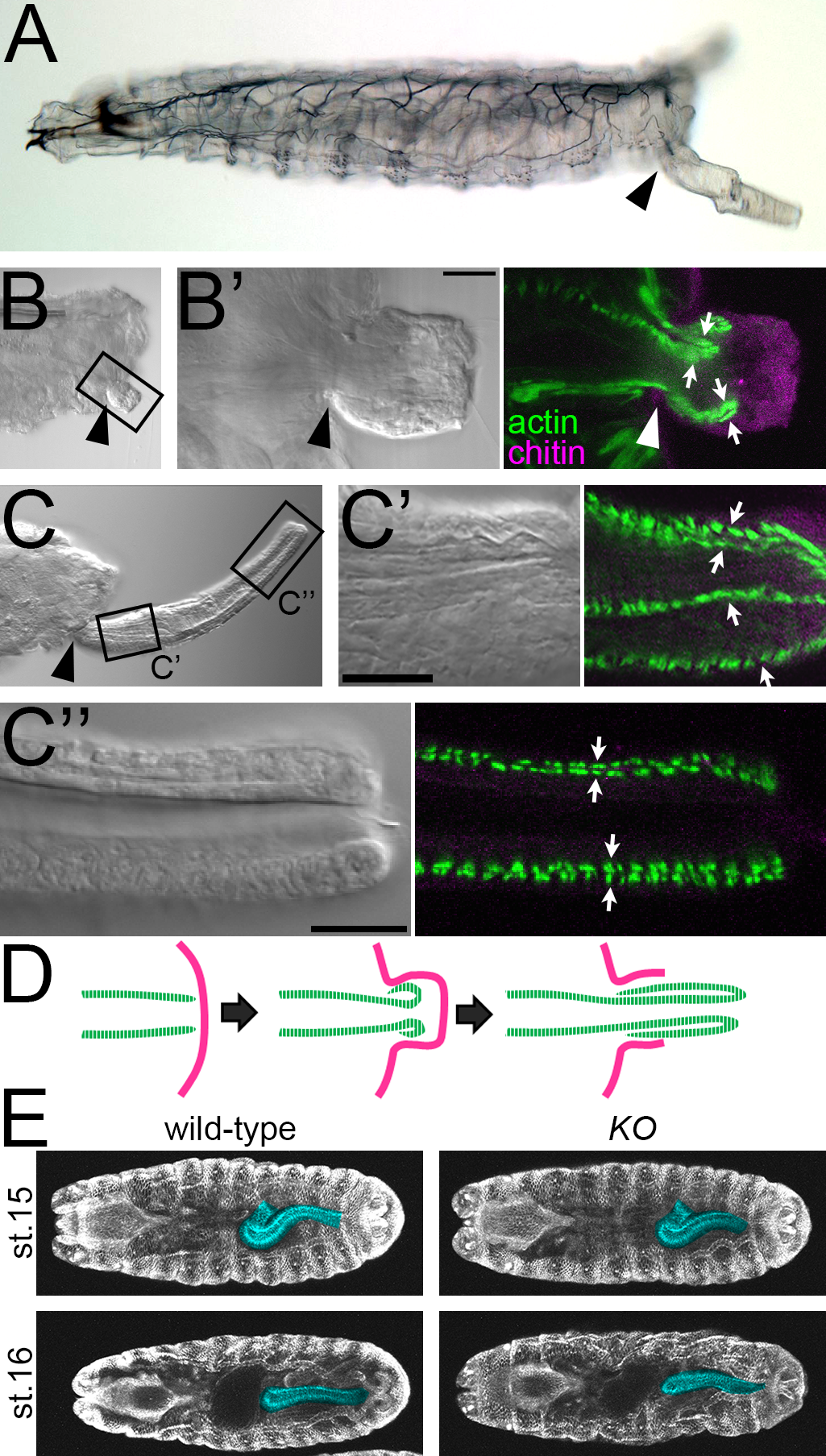

Supplement: S8 Fig — (A) A first instar obst-EKO/ obst-EDel larva. (B, C) The posterior parts of first instar obst-EKO homozygous larvae. Boxed regions are magnified in B’, C’ and C”. The gut musculature is visualized by phalloidin (green), and cuticle is stained by Fluostain (magenta). Arrowheads, anuses; arrows, doubled gut walls. (D) A model of how the hindgut protrudes to form the “doubled wall” morphology. Green, hindgut walls; magenta, cuticle. (E) Wild-type and obst-EKO homozygous embryos stained by anti-Fasciculin III antibody. Hindguts are pseudocolored in cyan. (TIF) [file pgen.1006548.s008.tif]

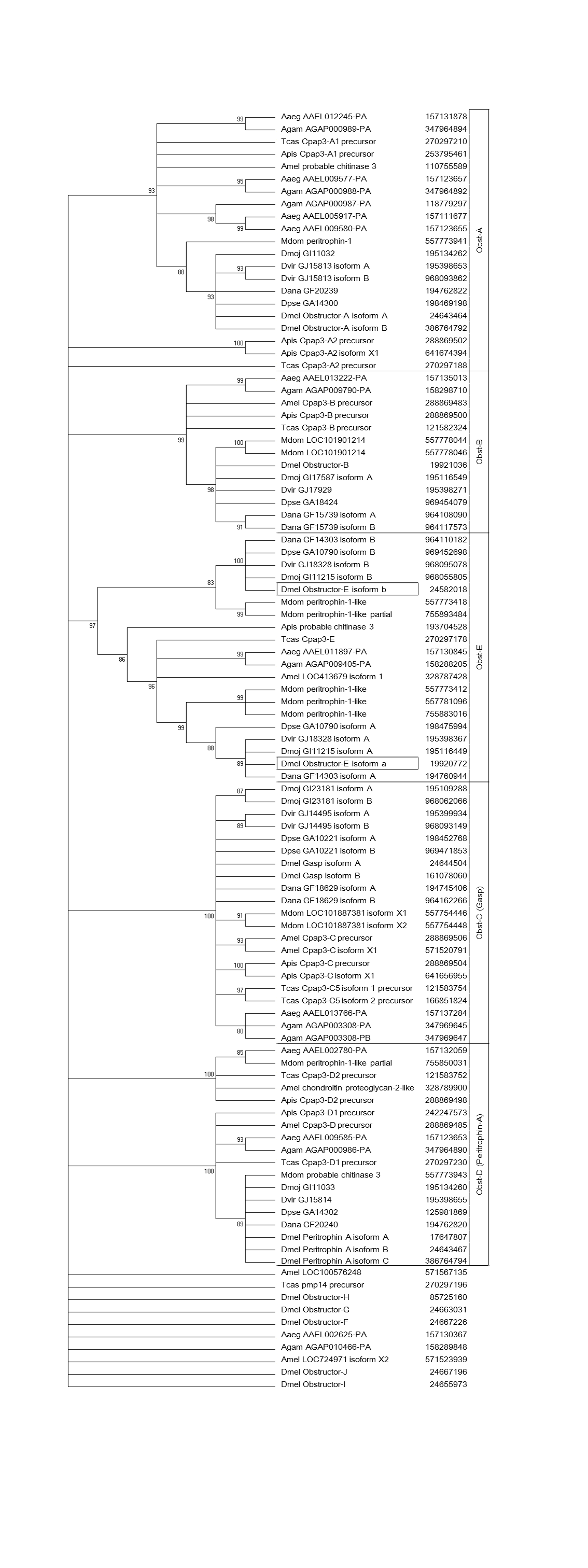

Supplement: S9 Fig — Phylogenetic analysis was done by MEGA5 [44] using the JTT cost matrix and complete deletion of indels. Bootstrap support is based on 1000 resampled data sets. Branches corresponding to partitions reproduced in less than 80% bootstrap replicates are collapsed. Dmel, Drosophila melanogaster; Dana, Drosophila ananassae; Dpse, Drosophila pseudoobscura; Dmoj, Drosophila mojavensis; Dvir, Drosophila virilis; Mdom, Musca domestica (housefly); Agam, Anopheles gambiae (malaria mosquito); Aaeg, Aedes aegypti (yellow fever mosquito); Tcas, Tribolium castaneum (red flour beetle); Amel, Apis mellifera (European honey bee); Apis, Acyrthosiphon pisum (pea aphid). 8–9 digit numbers on the right are NCBI GI numbers. (TIF) [file pgen.1006548.s009.tif]

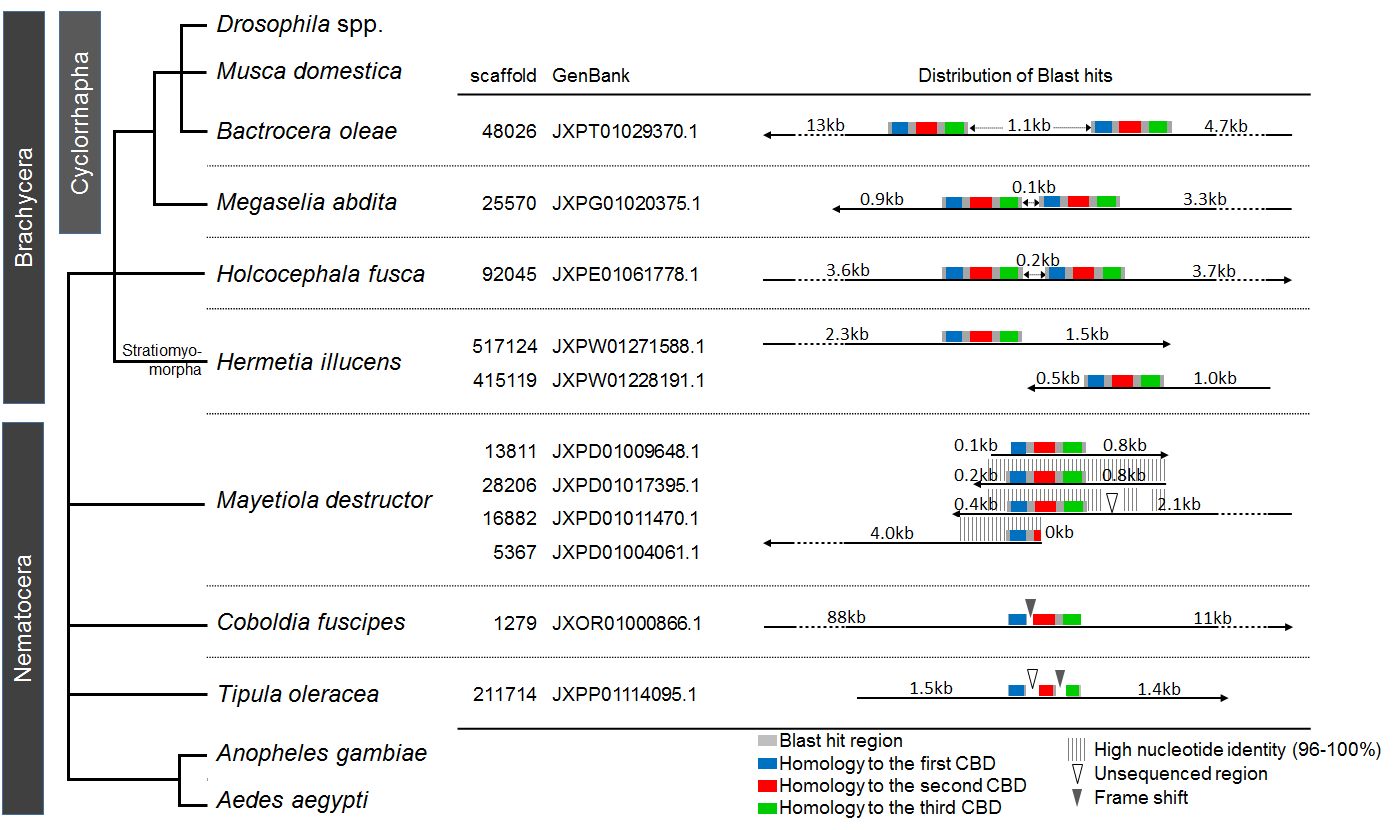

Supplement: S10 Fig — (Left) The phylogenic tree of the species examined, according to [15] and [19]. (Middle) The scaffold numbers and GenBank IDs of genomic sequences on which putative obst-E orthologs/variants were found by reciprocal blast searches with D. melanogaster. (Right) The distribution of the blast hit(s) on each genomic scaffold. (TIF) [file pgen.1006548.s010.tif]
